# Atg2 coordinates microbial metabolite signaling and epigenetic remodeling to maintain intestinal lipid homeostasis in *Drosophila*

**DOI:** 10.1186/s40168-026-02356-2

**Published:** 2026-03-27

**Authors:** Ping Wang, Xinran Li, Jiangong Zhang, Jiewei Wang, Li Hua Jin

**Affiliations:** 1 College of Life Sciences, Northeast Forestry University, Harbin, China; 2 College of Food and Biological Engineering, Qiqihar University, Qiqihar, China

**Keywords:** Atg2, *Drosophila*, Gut dysbiosis, SCFAs, Lipid metabolism, Innate immunity, Epigenetic regulation

## Abstract

**Background:**

The autophagy-related protein Atg2 maintains intestinal homeostasis by preventing lipid accumulation and microbial dysbiosis; however, the mechanisms by which these pathologies interconnect remain unknown.

**Results:**

We identify a microbiota‒metabolite‒epigenome axis through which *Atg2* deficiency triggers metabolic‒immune cascades in the *Drosophila* intestine. Tissue-specific *Atg2* depletion disrupts autophagic flux, provoking commensal overgrowth and pathogenic overproduction of short-chain fatty acids (SCFAs). Elevated SCFAs drive acetyl-CoA overflow, inducing global protein hyperacetylation that simultaneously activates lipogenic programs and primes innate immunity. Crucially, microbiota ablation or SCFAs restriction fully reverses lipid–immune dysregulation, mechanistically linking microbial metabolites to host pathophysiology.

**Conclusions:**

Our work establishes Atg2 as a guardian of microbiota-derived metabolite signaling, demonstrating that autophagy constrains microbial byproducts to prevent acetyl-CoA-mediated epigenetic hijacking of metabolic and immune networks. These findings reveal protein acetylation as a convergent regulator linking commensal ecology to host physiology, suggesting metabolite-centric therapies for dysbiosis-associated disorders.

Video Abstract

**Supplementary Information:**

The online version contains supplementary material available at https://doi.org/10.1186/s40168-026-02356-2.

## Background

Obesity is a chronic metabolic disorder characterized by pathological adipose tissue expansion and has emerged as a global pandemic, affecting 890 million adults worldwide in 2022 [1]. In addition to its role as a risk factor, obesity directly contributes to life-threatening comorbidities, including cardiovascular disease, type 2 diabetes mellitus, and nonalcoholic fatty liver disease (NAFLD) [2, 3]. Mounting evidence positions gut microbiome dysbiosis as a critical driver of metabolic dysfunction [4]. Clinical studies revealed that the enrichment of obesogenic taxa such as *Prevotella* [5] and steatosis-associated *Rickettsiaceae* and *Alteromonadales* increased in obese cohorts [6]. Although the microbiota impacts the metabiotic processes of the host, most of the mechanisms involved in this crosstalk remain elusive.

Gut microbiota-derived metabolites serve as pivotal mediators of host‒microbe communication, with short-chain fatty acids (SCFAs), bile acid intermediates, amino acid derivatives, and membrane-associated lipids constituting major functional classes [3, 7]. Among these microbial metabolites, SCFAs—primarily acetate, propionate, and butyrate—have emerged as the most extensively characterized and are produced through anaerobic fermentation of dietary fibers mediated by *Prevotella* species and other gut microbiota [8]. Recent work has demonstrated their ability to rescue phospholipid biosynthesis in gnotobiotic mice [9], highlighting essential roles in maintaining metabolic homeostasis. SCFAs can regulate host chromatin through histone posttranslational modifications (PTMs), thereby dynamically modulating enzyme activity [10]. At the epigenetic level, butyrate-derived acetyl-CoA dynamically regulates chromatin states through two mechanisms: ACLY-dependent histone acetylation via HAT activation and direct HDAC inhibition [11]. Despite these advances, critical gaps persist in understanding how microbial metabolite gradients are transduced into spatially resolved epigenetic signatures, particularly within stem cell niches where metabolite availability intersects with immune signaling cascades.

Autophagy, an evolutionarily conserved lysosomal degradation pathway, mediates the turnover of cytoplasmic components, including long-lived proteins, lipid droplets, and damaged organelles [12]. Pioneering studies in yeast established Atg2 as a critical autophagy effector that is essential for both autophagosome biogenesis and the cytoplasm-to-vacuole targeting pathway [13, 14]. Subsequent work revealed that yeast Atg2 localizes to phagophore assembly sites via interaction with Atg18, facilitating membrane expansion during autophagosome formation [15]. Human Atg2 transports phosphatidylethanolamine (PE) from the endoplasmic reticulum to grow autophagosome membranes in HeLa cells, and this lipid transfer function is conserved in mammals [16, 17]. These findings demonstrate that Atg2 preferentially mobilizes short-chain fatty acyl PE species to maintain lipid droplet homeostasis in *Drosophila* [18]. Despite these advances, the tissue-specific roles of Atg2 in coordinating autophagy, lipid metabolism, and intestinal physiology remain poorly defined, particularly in stem cell-driven epithelial systems where dynamic lipid mobilization intersects with microbial and metabolic regulation.

*Drosophila melanogaster* has emerged as a powerful model organism for dissecting triacylglycerol (TAG) homeostasis and lipid-associated pathologies owing to its conserved metabolic pathways, organ system homology, and functional overlap of disease-related genes with those of humans [19, 20]. Central to these studies is the *Drosophila* midgut—a functionally compartmentalized organ divided into five anatomically distinct regions, each specialized for nutrient processing, absorption, or microbial interaction [21, 22]. This intestinal epithelium, which is maintained by intestinal stem cells (ISCs) and enteroblasts (EBs), harbors a simplified yet representative microbiome dominated by *Lactobacillaceae* and *Acetobacteraceae* species, enabling precise interrogation of host‒microbe crosstalk [22, 23].

Here, we revealed a novel role for the autophagy-related protein Atg2 in coordinating intestinal metabolic homeostasis in *Drosophila*. Through tissue-specific genetic manipulation of ISCs/EBs, we demonstrated that *Atg2* depletion disrupts a triad of critical processes: (1) SCFAs overproduction, which drives lipid accumulation; (2) dysbiosis, which triggers IMD/Toll-mediated immunity; and (3) SCFAs-dependent collapse of EGFR/JNK/JAK-STAT proliferative signaling. Our multiomics analyses revealed that Atg2 functions as a metabolic‒epigenetic integrator, linking microbial SCFAs production to acetyl-CoA-fueled chromatin remodeling at loci that govern lipogenesis (*FASN* and *ACSS*) and immune activation (*Relish*). By elucidating this autophagy‒microbiota‒metabolism axis, our work redefines the role of Atg2 beyond its role in canonical lipid transport, positioning it as a central regulator of microbial metabolite signaling with direct implications for dysbiosis-associated metabolic disorders.

## Methods

### Animals and cell lines

Flies were maintained at 25°C under 60% humidity and a 12-h light/dark cycle on standard cornmeal-yeast medium (SM). For progenitor-specific manipulation, *esg*^*ts*^*-GAL4 UAS-GFP* and *Da*^*ts*^*-GAL4* lines were crossed with RNAi or overexpression strains at 22°C. Adult F1 females were shifted to 29°C for 7 days to induce transgene expression. Three independent *Atg2* RNAi lines and *Atg2* mutant were validated. The genotypes are listed in Table S1. The *Atg2* RNAi THU3698 line (hereafter referred to as *Atg2*^*RNAi*^) was selected for subsequent experiments due to its superior gene silencing efficacy and potent antiproliferative effects in validation assays.

The *Drosophila* S2 cell line was cultured in Schneider’s medium (Gibco 21720024) supplemented with 10% heat-inactivated fetal bovine serum (FBS, Gibco A5670701) and 0.1% cell culture antibiotics (VivaCell C3420-0100) at 25 °C.

### Germ-Free (GF) fly generation

To generate axenic flies, adult females were maintained in sterile vials for 7 days on filter discs soaked in 5% sucrose containing a combined antibiotic regimen [100 μg/mL ampicillin (Biotopped A6180F), 50 μg/mL tetracycline (Biotopped T9029F), 100 μg/mL kanamycin (Solarbio K8020), and 10 μg/mL erythromycin (Biotopped E6100F)] [24–27]. This treatment did not adversely affect *Drosophila* stem cell activity, lipid metabolism, or immunity. Successful microbiota depletion was verified by 16S rRNA qPCR using primers 338F/806R.

Adult flies were transferred to fresh apple juice agar plates supplemented with yeast paste. Following a 1-day habituation period, flies were allowed to lay eggs for 4 h. The collected eggs were gently brushed into PBS and subjected to surface sterilization by immersion in 50% bleach for 2 min, followed by two washes in 70% ethanol for 1 min each and three rinses in sterile deionized water. The sterilized eggs were then transferred to sterile fly vials containing autoclaved food. To confirm successful removal of bacterial contaminants, adult flies from each experimental group were homogenized and plated on MRS agar for microbial testing after the experiment.

### Mono-association of bacteria

For bacterial mono-association, *Lactobacillus plantarum* was cultured overnight at 37°C in 20 mL of MRS broth. A 50 μL aliquot of the culture (OD₆₀₀ = 0.2) was applied onto the surface of sterilized fly food. The inoculated medium was allowed to absorb for 30 min at room temperature before introducing the GF flies.

### Chemical treatments

The flies received filter discs containing antibiotics alone, antibiotics with 50 mM sodium acetate (Sigma-Aldrich 127-09-3), antibiotics with 25 mM calcium propionate (Sigma-Aldrich 4075-81-4), or antibiotics with 25 mM sodium butyrate (Sigma-Aldrich 156-54-7). Fresh solutions were replaced daily. After 72 h, the midguts were dissected for analysis.

The flies were raised and maintained on SM with histidine (Macklin CAS71-00-1, SM with 1 g/L histidine or 4 g/L histidine).

For trichostatin A (TSA, CAYMAN 58880-19-6) treatment, an inhibitor of class I/II histone deacetylases, TSA was dissolved in DMSO (Sigma 67-68-5). At the organismal level, adult flies were orally administered 10 μM TSA using absorbent discs for 48 h (acute treatment) prior to immunofluorescence staining. At the cellular level, S2 cells were treated with 0.2 μg TSA or DMSO, and the cells were harvested 24 h after treatment for ChIP (Chromatin immunoprecipitation)-qPCR analysis.

For chloroquine (CQ, Aladdin C91834) treatment, an inhibitor of autophagy, CQ was dissolved in DMSO. Adult flies were orally administered 1 mM CQ using absorbent discs for 48 h before immunofluorescence staining. In parallel, S2 cells were treated with 100 nM CQ or DMSO, and cells were harvested 24 h after treatment for ChIP-qPCR analysis.

### Lifespan and gut permeability assays

Age-synchronized flies were maintained on 5% sucrose agar. Mortality was recorded daily. Gut leakage was assessed via the Smurf assay: starved flies consumed FD&C Blue No. 1 dye (Sigma-Aldrich 3844-45-9, 2.5% in agar), and leakage was scored after 9 h.

### Histology and imaging

The midgut was fixed in 4% formaldehyde, blocked with 0.5% goat serum, and stained overnight with primary antibodies. After secondary antibody incubation and Hoechst counterstaining, the samples were imaged on a Zeiss Axioskop 2 Plus. For FASN staining, standard fixation was followed by methanol dehydration to improve antibody penetration. The following primary antibodies were used: mouse anti-GFP (Thermo Fisher MA5-54334, 1:200), rabbit anti-phospho-histone H3 (Millipore H0412, 1:200), mouse anti-FASN (Santa Cruz Biotechnology sc55580, 1:50), and mouse anti-Ac-lysine (Santa Cruz Biotechnology sc32268, 1:50).

Lipid droplets were visualized via Nile Red (Sigma-Aldrich 7385-67-3, 0.1 ng/mL). Midguts from adult females were dissected in ice-cold PBS (Servicebio G4202) and fixed in 4% paraformaldehyde (Sigma-Aldrich 50-00-0) for 30 min at 25 °C. After three 5-min washes with PBS, the tissues were costained with Nile red (0.1 ng/mL in PBS/30% glycerol) and Hoechst (Sigma-Aldrich 23491-45-4, 1:500) for 60 min in the dark. Unbound dye was removed through three additional 5-min washes with PBS. The samples were mounted in 70% glycerol-based (Sigma-Aldrich G7793) antifade medium and imaged immediately to prevent fluorophore quenching.

### Transcriptomic and microbiome analysis

RNA was extracted from 50 midguts per replicate using TRIzol (Invitrogen, 15596026CN). Libraries were sequenced on the Illumina NovaSeq X Plus and DNBSEQ-T7 platforms (Majorbio) by Majorbio BioPharm Technology Co., Ltd. (Shanghai, China). DEGs were identified via DESeq2 (|log2FC|> 0.58, padj < 0.05). For 16S sequencing, midgut DNA from 20 midguts per replicate was amplified with 341F/806R primers and sequenced on a NovaSeq 6000 by Majorbio BioPharm Technology Co., Ltd. (Shanghai, China). The raw reads were processed via the Majorbio Cloud Platform (v2.6).

### SCFAs analysis

The concentrations of SCFAs were determined using gas chromatography–mass spectrometry (GC-MS). Analysis was performed on a Shimadzu QP2020 system by Biotree Biotech Co., Ltd. (Shanghai, China) following the manufacturer’s standard protocol. SCFAs were quantified by the external standard method, and all reported concentrations were normalized to the corresponding sample weight.

### quantitative PCR (qPCR)

Total RNA was extracted using TRIzol Reagent. The RNA was reverse-transcribed via M-MLV reverse transcriptase (Promega M5301). SYBR GreenER (Invitrogen 11,762,500) and *rp49* normalization were used for qPCR (primers in Table S2 and S3).

### ChIP-qPCR

ChIP assay was performed using a commercial ChIP Assay Kit (Beyotime P2078). Briefly, *Drosophila* S2 cells (3 × 10^7^) were fixed with 1% formaldehyde, and cross-linking was quenched by adding glycine to a final concentration of 125 mM. The chromatin was then isolated, sheared, and a 20 μL aliquot was reserved as the input control. The remaining chromatin was incubated overnight with specific antibodies, followed by immunoprecipitation using a mixture of Protein A/G agarose beads. The antibodies used were anti-H3K27ac (Abcam ab4729) and normal mouse IgG (Santa Cruz Biotechnology sc-515946) as a negative control. After reversing the cross-links, the purified DNA was analyzed by qPCR with SYBR GreenER or by standard PCR. For ChIP-qPCR, the relative enrichment of target regions was expressed as fold change over the IgG control. Primer sequences used for ChIP-qPCR are listed in Table S4.

### Enzyme assays

Acetyl-CoA carboxylase (ACC) and ATP citrate lyase (ACLY) activities were quantified via an insect acetyl-CoA carboxylase ELISA kit (Nanjing Jiancheng Bioengineering Institute H232-1-2) and an insect ATP citrate lyase ELISA kit (Nanjing Jiancheng Bioengineering Institute H478-1) using fly homogenates. Standard curves were included per plate (R^2^ > 0.99). Enzyme activities were normalized to weight.

### Triglyceride (TG) and Non-Esterified Fatty Acid (NEFA) assay

TG levels were measured using a Triglyceride assay kit (Nanjing Jiancheng Bioengineering Institute A110-2-1), and absorbance was recorded at 500 nm. NEFAs were quantified using a corresponding assay kit (Nanjing Jiancheng Bioengineering Institute A042-1-1), with absorbance measured at 440 nm. TG and NEFA levels were normalized to sample weight.

### Statistics

Individual data points in all graphical representations represent biological replicates (n ≥ 3) with standard mean errors (SEMs). Digital quantifications were performed via Fiji (ImageJ v2.3.0/1.53q). The working model was created with BioGDP.com. Unpaired t tests (GraphPad Prism) were used to determine significance: *p < 0.05, **p < 0.01, ***p < 0.001, and “ns” denotes no significant difference.

## Results

### Atg2 deficiency-induced altered lipid metabolism depends on the gut microbiota in the *Drosophila* midgut

**Fig. 1:**
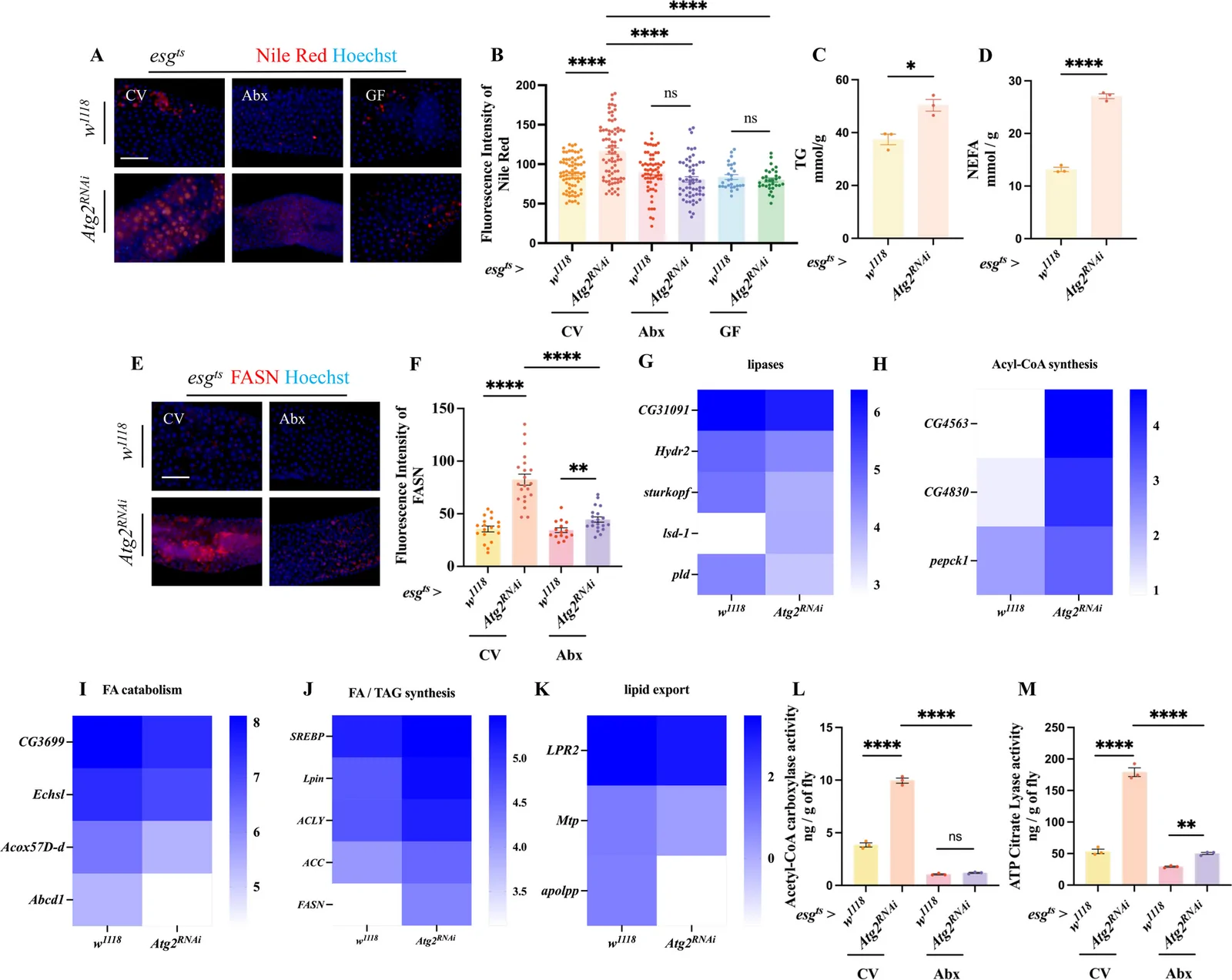
Atg2 regulates intestinal lipid metabolism in a gut microbiota-dependent manner. **A-B** Nile Red staining of neutral lipids in control and *Atg2*-knockdown flies under conventional (CV), antibiotic-treated (Abx), or germ-free (GF) conditions. Hoechst (blue) labels nuclei. **A** Representative view of the AMG. **B** Quantification of fluorescence intensity. Each point represents one fly from three biological replicates. **C** Measurement of TG levels from three independent experiments. **D** Measurement of NEFA levels from three independent experiments. **E–F** Immunostaining and quantification of FASN. Hoechst (blue) labels nuclei. **C** Representative transverse views of the AMG. **D** Quantification of fluorescence intensity. Each point represents one fly from three biological replicates. **G-K** Heatmaps showing transcript levels of genes involved in lipid metabolism. Data are from three independent experiments. **L** Quantification of ACC enzyme activity from three independent experiments. **M** Quantification of ACLY enzyme activity from three independent experiments. Scale bars represent 50 μm. The error bars represent the SEMs. Student’s t tests, *p < 0.05, **p < 0.01, ***p < 0.001, ****p < 0.0001, and NS (nonsignificant) represent *p* > 0.05

Previous studies have established conserved roles for Atg2 proteins in autophagosome formation and lipid metabolism across species [17, 18]. However, the precise mechanistic roles of Atg2 in modulating lipid homeostasis in the *Drosophila* midgut remain incompletely characterized. Therefore, we targeted *Atg2* knockdown specifically in intestinal progenitor cells via *esg*^*ts*^-*GAL4*-driven *UAS-Atg2* RNAi transgenic flies and performed mRNA sequencing (mRNA-seq). RNA sequencing analysis revealed that 469 genes were upregulated, and 456 genes were downregulated in the *Atg2*-deficient flies (*p* < 0.05 and fold change > 1.5) compared with the control flies (Fig. S1A). Notably, the DEGs were clustered in lipid biosynthesis pathways, immune response pathways, epigenetic regulation modules, and cell proliferation-related genes (Fig. S1A). KEGG pathway analysis confirmed that knocking down *Atg2* promoted the expression of genes involved in metabolic pathways (Fig. S1B). Consistent with the increase in lipogenic gene expression, Nile red staining revealed pronounced neutral lipid accumulation in the anterior midgut (AMG) of the *Atg2*-deficient flies. In addition, control midguts presented spared and small cytoplasmic lipid droplets (LDs), whereas *Atg2*-deficient intestines presented increased LDs sizes (Fig. 1A-B).

To further characterize lipid metabolic changes, levels of TG and NEFA were measured. Both TG and NEFA levels were significantly elevated in *Atg2*-deficient flies compared with controls (Fig. 1C–D). To determine the efficiency of Atg2 knockdown, we verified that the mRNA level decreased significantly (Fig. S1C). Notably, this phenotype was consistently recapitulated using two additional independent RNAi lines, confirming the specificity of the observed effects (Fig. S1D-E). To further validate the causal relationship between *Atg2* loss and the observed phenotypes, we employed an independent *Atg2* mutant to exclude potential off-target effects. Consistent with our initial findings, this genetic model also showed significant lipid accumulation (Fig. S1F-G). Additionally, we found that *Atg2* overexpression induced by *esg*^*ts*^*-GAL4* significantly rescued lipid accumulation (Fig. S1H-I), establishing a direct causal relationship between *Atg2* depletion and cellular lipid accumulation. Lipid accumulation induced by *Atg2* depletion was alleviated by rapamycin treatment (Fig. S1H-I). FASN, a key enzyme for TAG synthesis, was significantly upregulated when *Atg2* was knocked down (Fig. 1E-F). In addition, Atg2 simultaneously suppresses lipid catabolic/export enzymes and activates lipogenic pathways, revealing a dual regulatory role of Atg2 in lipid metabolism (Fig. 1G-K). These transcriptional changes were corroborated by functional assays showing increased enzymatic activity of key lipid synthesis regulators: ACC (Fig. 1L) and ACLY (Fig. 1M). We investigated whether the increased volume of LDs in *Atg2*-depleted flies was simply due to a defect in lipophagy or was a specific phenotype associated with *Atg2* depletion. Immunostaining for p62 (SQSTM1), a well-characterized autophagy receptor that accumulates when autophagic flux is impaired, provides definitive cytological evidence. The marked increase in p62 puncta within progenitor cells upon *Atg2* knockdown directly confirms a functional autophagy defect in these cells (Fig. S1J-K). RNAi-mediated knockdown of the core autophagy regulators Atg1, Atg8a, Atg10, and Atg16, essential for autophagosome formation, recapitulated the lipid dysregulation phenotype, resulting in significant increases in the LDs volume and number compared with those of the controls (Fig. S1L-M).

Given the established crosstalk between intestinal microbes and host metabolism [28], we investigated whether microbiota depletion via antibiotic cocktail (Abx) treatment could ameliorate *Atg2* RNAi-induced lipid accumulation. Remarkably, Abx-treated *Atg2*-deficient flies showed complete normalization of LDs size. The small LDs in the Abx-treated flies indicated that antibiotic treatment was effective (Fig. 1A-B). This metabolic rescue correlated with restored enzymatic activity levels. FASN (Fig. 1E-F), ACC (Fig. 1L), and ACLY (Fig. 1M) levels were not significantly different between the Abx-treated *Atg2* RNAi and control groups. To further support our conclusions, we validated the key findings using a GF fly model. The lipid accumulation was significantly alleviated in GF flies following *Atg2* depletion (Fig. 1A-B). These findings establish that Atg2 maintains lipid homeostasis in the midgut through mechanisms intrinsically linked to the microbial community.

### Atg2 deficiency induces microbial dysbiosis and impacts host lifespan

**Fig. 2:**
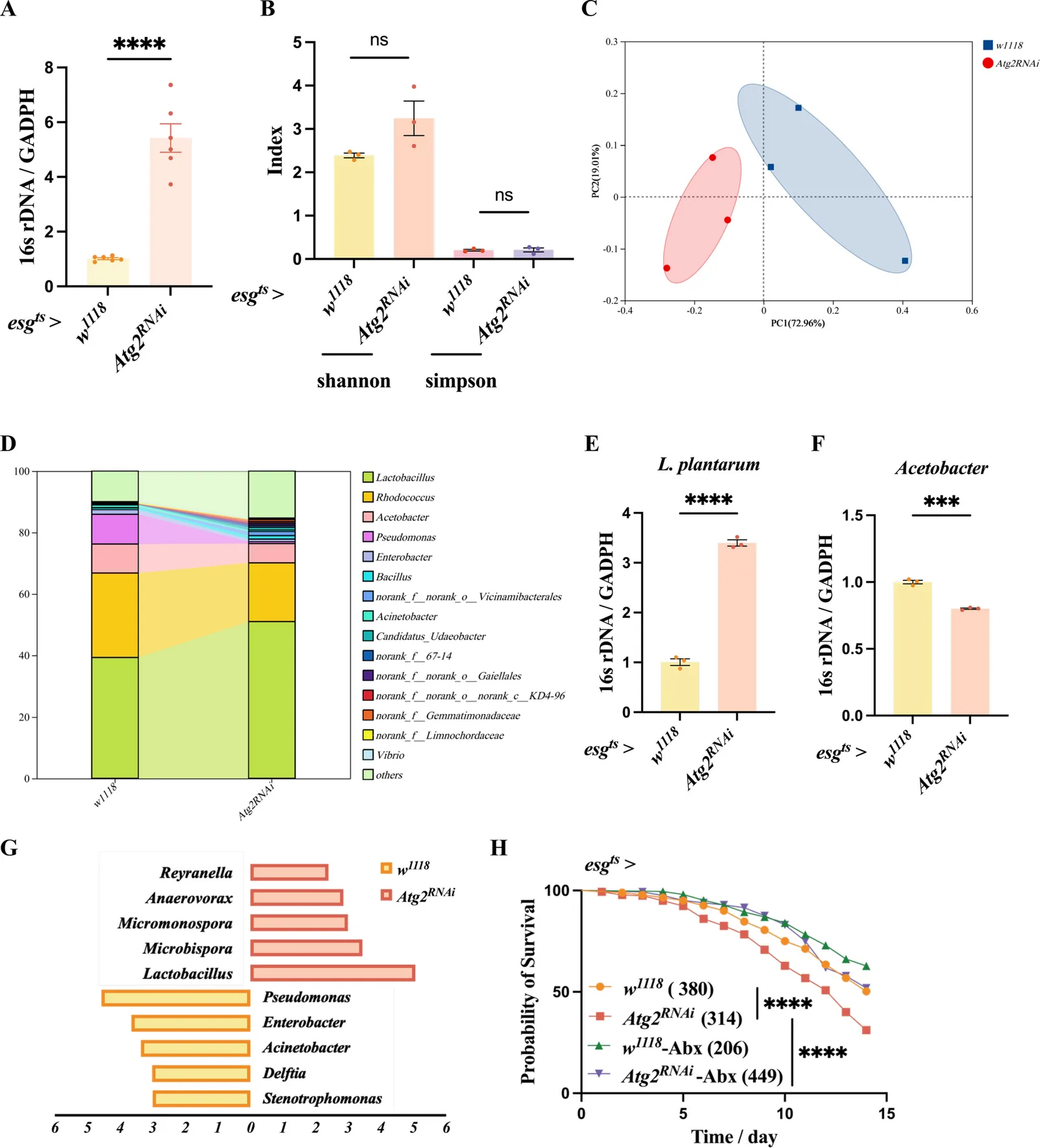
Loss of Atg2 alters the gut microbiota composition. **A** Gut microbiota loading was determined by qPCR from three independent experiments. **B** α diversity in the gut samples. Shannon and Simpson diversity analyses of the bacterial species in the gut samples were performed via the Wilcoxon rank‐sum test. **C** Principal coordinate analysis (PCoA) analysis showing significant differences in the gut microbiota via the Bray–Curtis test. **D** Relative abundance of the taxonomic classifications at the genus level in intestinal samples. **E** *L. plantarum* loading determined via qPCR from three independent experiments. **F** *Acetobacter* loading was determined by qPCR from three independent experiments. **G** LEfSe analysis of significant bacteria whose relative abundance differed between the intestinal samples (*P* < 0.05). **H** Survival analysis of control and *Atg2*-deficient flies with or without antibiotic treatment (Abx). Statistical significance was determined by the log-rank test. The error bars represent the SEMs. Student’s t tests, *p < 0.05, **p < 0.01, ***p < 0.001, ****p < 0.0001, and NS (nonsignificant) represent *p* > 0.05

To investigate whether intestinal microbes are affected by *Atg2* depletion, the total bacterial load was first quantified. Compared with control flies, *Atg2*-deficient flies presented a marked increase in microbial colonization (Fig. 2A).

Therefore, we investigated the intestinal microbial composition. 16S rRNA sequencing revealed no significant differences in α-diversity metrics (Fig. 2B); however, β-diversity analysis via principal coordinates (PCoA) demonstrated distinct clustering of microbial communities between *Atg2*-deficient and control flies (Fig. 2C). Taxonomic profiling revealed specific compositional shifts. The *Atg2*-deficient guts presented increased relative abundances of *Lactobacillus* and *Enterobacter* and decreased proportions of *commensal Acetobacter*, *Rhodococcus*, *and Pseudomonas* at the genus level (Fig. 2D-F). These observations indicate that decreased *Atg2* levels result in alterations in the intestinal microbiota. Notably, we observed the expansion of potentially pathogenic genera, including *Microbispora*, *Micromonospora*, and *Anaerovorax*, which were more abundant in *Atg2*-deficient flies (Fig. 2G).

Given the established links between microbial dysbiosis and host mortality [29], we next examined lifespan. Consistent with increased bacterial loads, *Atg2*-deficient flies presented a shorter median lifespan than controls did (Fig. 2H). Notably, the observed *Lactobacillus* expansion in the *Atg2*-deficient flies aligns with established pathogenic outcomes in *Drosophila* (Fig. 2D-E, 2G). As demonstrated [30], *Lactobacillus*-dominated microbiomes reduce lifespan (Fig. 2H). Crucially, antibiotic treatment completely rescued this phenotype (Fig. 2H), indicating that the *Atg2* depletion-mediated reduction in longevity was microbiota dependent. These data collectively demonstrate that Atg2 maintains intestinal homeostasis by restraining pathogenic microbial expansion, with important effects on host survival.

### SCFAs-mediated metabolic defects underlie* Atg2* deficiency-induced lipogenesis

**Fig. 3:**
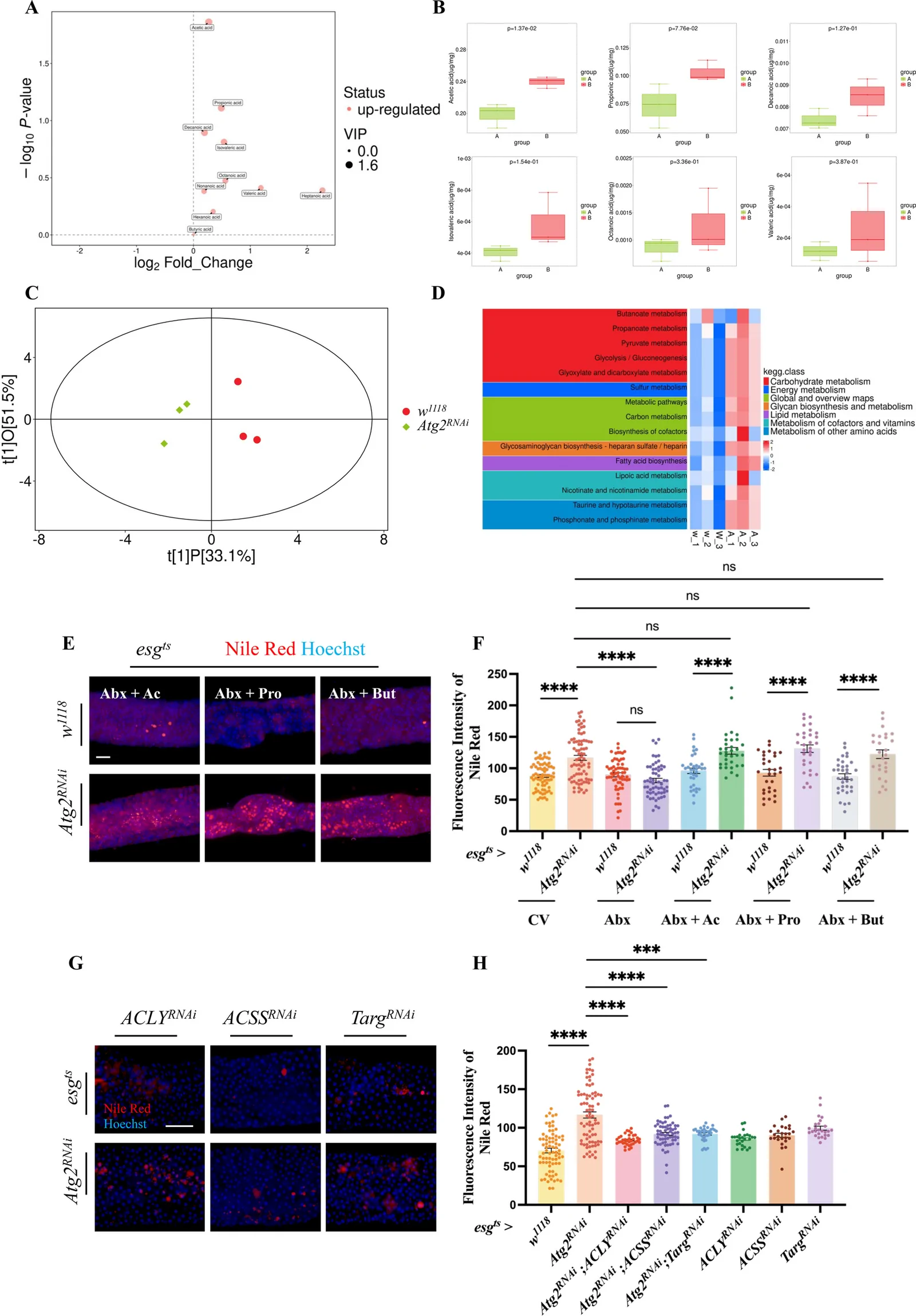
Atg2 regulates intestinal lipid metabolism in a gut metabolite-dependent manner. **A** Volcano plot illustrating the differential abundance of SCFAs between control and *Atg2*-knockdown flies. **B** Quantification of SCFAs levels from three independent experiments. A represents control, and B represents *Atg2*-knockdown flies. **C** OPLS-DA score plot derived from metabolomic profiles of control and *Atg2*-knockdown flies. **D** KEGG enrichment analysis of metabolites altered by *Atg2* knockdown. **E–F** Effect of sodium acetate (Ac), sodium propionate (Pro), and sodium butyrate (But) supplementation on intestinal lipid accumulation, visualized by Nile Red staining. Hoechst (blue) labels nuclei. **E** Representative view of the AMG. **F** Quantification of fluorescence intensity. Each point represents one fly from three biological replicates. **G‒H** Nile Red staining of lipids in Atg2-deficient flies following knockdown of *ACLY*, *ACSS*, and *Targ* in ISCs/EBs. **G** Representative views of the AMG. **H** Quantification of fluorescence intensity. Each point represents one fly from three biological replicates. Scale bars represent 50 μm. The error bars represent the SEMs. Student’s t tests, *p < 0.05, **p < 0.01, ***p < 0.001, ****p < 0.0001, and NS (nonsignificant) represent *p* > 0.05

Mounting evidence has established the gut microbiota as a key modulator of host lipid metabolism, particularly through bacterial fermentation-derived metabolites such as SCFAs [31]. Consistent with this paradigm, our analysis revealed that *Atg2* deficiency remodeled the intestinal microbiome, enriching obesity-associated taxa, including *Christensenellaceae* (Fig. S2A) and *Pasteurellaceae* (Fig. S2A). Notably, these microbial shifts mirror clinical observations linking elevated fecal SCFAs levels to adiposity in overweight individuals [31]. Mechanistically, *Atg2* loss induces profound dysbiosis characterized by the expansion of SCFAs-hyperproducing genera [32]: *Prevotella* (Fig. S2A-B), *Streptococcus* (Fig. S2A, S2C), *Eubacterium* (Fig.S2A), and *Lactobacillus* (Fig. 2D, S2D). Moreover, mono-association with *L. plantarum* was sufficient to recapitulate the dysbiosis-induced lipid accumulation phenotype (Fig. S2E-F), providing direct evidence for a microbial contribution to the observed metabolic perturbation. Concurrently, we observed depletion of *Rikenellaceae* (Fig. S2A), a taxon inversely correlated with SCFAs levels [33]. To directly examine the potential impact of *Atg2* depletion on SCFAs production, we measured the levels of various SCFAs using GC–MS. Our analysis revealed a significant increase in SCFAs concentrations in *Atg2*-deficient flies compared with controls (Fig. 3A-B).

This metabolic shift was further supported by a clear separation in the OPLS-DA score plot (Fig. 3C), indicating a distinct metabolite profile. Consistently, KEGG pathway enrichment analysis revealed significant alterations in key metabolic pathways, including butanoate, propanoate, and pyruvate metabolism, as well as fatty acid biosynthesis (Fig. 3D). These findings collectively position Atg2 as a critical regulator of microbial ecology, with dysbiosis shifting, driving SCFAs overproduction, and subsequent metabolic dysregulation.

To link SCFAs to lipid accumulation, we administered propionate/acetate/butyrate to Abx-treated *Atg2* RNAi flies. SCFAs supplementation restored neutral lipid accumulation (Fig. 3E-F), implicating microbiota-derived SCFAs as drivers of lipogenesis. Mechanistically, SCFAs are converted to cytosolic acetyl-CoA via ACSS and ACLY [34]. We further hypothesized that acetyl-CoA pools are critical for microbe-mediated modulation of host lipid metabolism. Strikingly, intestine-*specific ACLS* and *ACLY* RNAi reduced LDs (Fig. 3G-H). In support of this metabolic axis, *silencing Targ* (monocarboxylate transporter) decreased lipid deposition (Fig. 3G-H), confirming that SCFAs uptake is rate-limiting for lipogenesis. These findings collectively demonstrate that *Atg2* deficiency remodels the gut microbial ecology to favor SCFAs hyperproduction, which fuels lipogenic programs through acetyl-CoA metabolic flux.

### SCFAs coordinate metabolic and epigenetic regulation through HDAC3

**Fig. 4:**
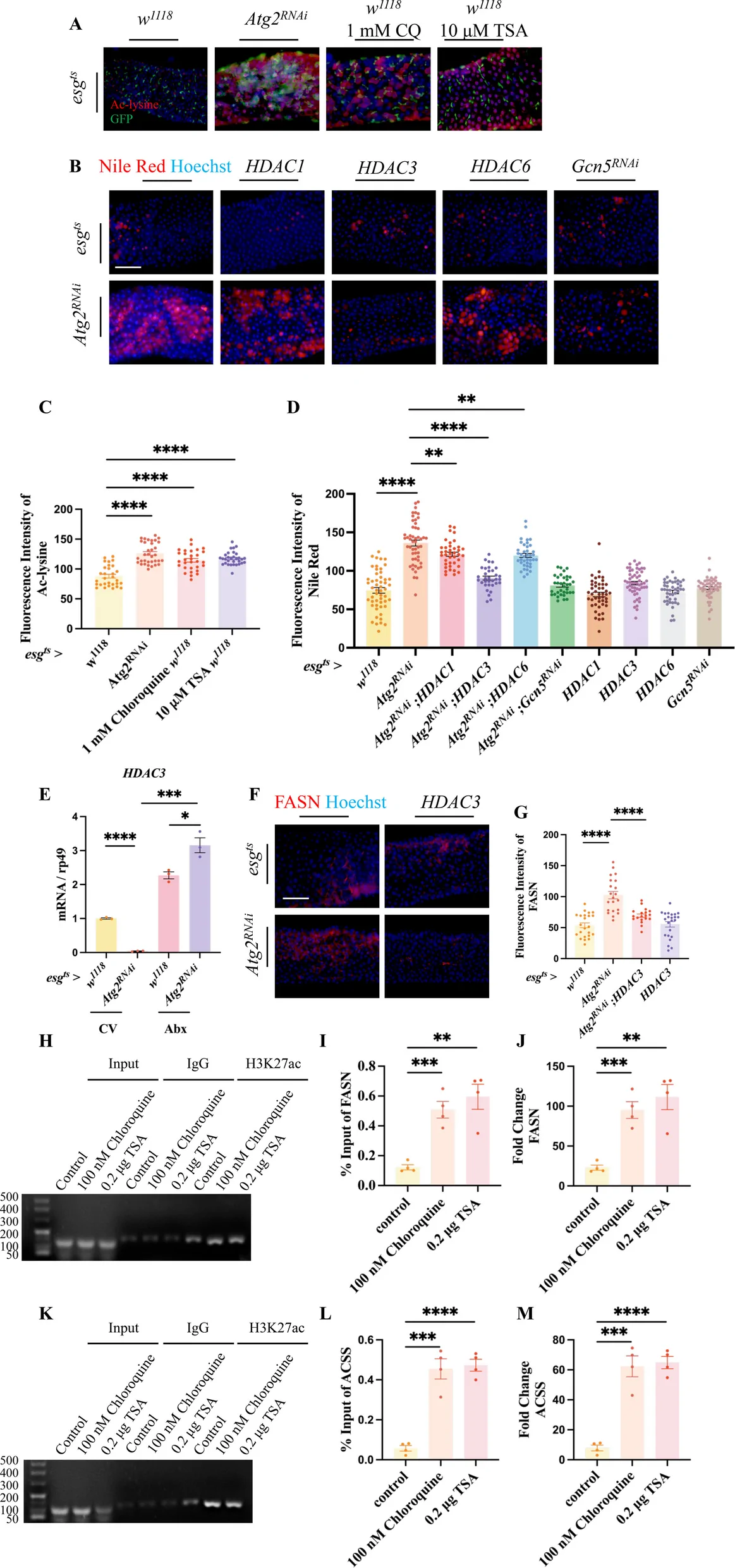
Atg2 regulates intestinal lipid metabolism via acetylation. **A** Assessment of protein acetylation levels by anti-acetylated lysine (Ac-lysine) immunostaining. Representative images show Ac-lysine (red) and GFP (green) colocalization.** B** Nile Red staining of neutral lipids in Atg2-deficient flies overexpressing *HDAC1*, *HDAC3*, or *HDAC6*, or with knockdown of *Gcn5*. Representative images of the AMG are shown. **C-D** Quantification of fluorescence intensity. Each point represents one fly from three biological replicates. **E** RT‒qPCR analysis of *HDAC3* expression in intestinal samples from three independent experiments. **F-G** FASN immunostaining in *Atg2*-deficient flies overexpressing HDAC3.** F** Representative images of the AMG. **G** Quantification of fluorescence intensity. Each point represents one fly from three biological replicates. **H-J** ChIP-PCR/qPCR analysis of H3K27ac enrichment at the *FASN* promoter locus from three independent experiments. **K-M** ChIP-PCR/qPCR analysis of H3K27ac enrichment at the *ACSS* promoter locus from three independent experiments. Scale bars represent 50 μm. The error bars represent the SEMs. Student’s t tests, *p < 0.05, **p < 0.01, ***p < 0.001, ****p < 0.0001, and NS (nonsignificant) represent *p* > 0.05

To determine the established role of SCFAs in modulating histone acetylation [25], we investigated how acetylation regulates intestinal lipid metabolism. Given the potential link between microbial metabolites and midgut epithelial protein acetylation, we observed a marked increase in lysine acetylation in *Atg2*-deficient flies, a phenotype that was phenocopied by CQ and TSA treatment (Fig. 4A, C).

Genetic ablation of the acetyltransferase Gcn5 abolished *Atg2* deficiency-induced pathological lipid accumulation (Fig. 4B, D), directly establishing Gcn5-mediated hyperacetylation as the mechanistic driver of lipotoxicity. Tissue-specific overexpression of *Drosophila* HDAC isoforms revealed isoform-specific functional divergence. Overexpression of *HDAC3* reduced neutral lipid accumulation and completely rescued FASN upregulation in *Atg2*-deficient midguts, whereas overexpression of HDAC1 or HDAC6 had mild effects (Fig. 4B, D). Next, through mechanistic studies, we demonstrated that *Atg2* deficiency suppresses *HDAC3* through dual pathways. First, *Atg2* loss reduced *HDAC3* transcript levels (Fig. 4E). Second, microbiota depletion via Abx treatment restored *HDAC3* expression in the mutants to wild-type levels, indicating microbial reinforcement of *HDAC3* suppression (Fig. 4E). *HDAC3* overexpression significantly suppressed *FASN* expression (Fig. 4F-G), demonstrating the indispensable role of chromatin remodeling in mediating microbiota-driven lipogenesis. To provide direct evidence of epigenetic reprogramming, we mapped H3K27ac at lipid biosynthesis. ChIP-PCR and ChIP-qPCR displayed robust enrichment at the *FASN* and *ACSS* promoters following treatment with CQ and TSA (Fig. 4H-M). These findings delineate a unified mechanism through which gut microbiota-derived SCFAs coordinate lipid biosynthesis via metabolic and epigenetic interplay.

### Histidine supplementation reverses dysbiosis-driven lipogenesis

Emerging clinical evidence positions histidine as a key regulator of hepatic steatosis, with circulating histidine levels showing a strong inverse correlation with lipid accumulation severity in humans and mice [6]. Histidine ammonia-lyase enzymes convert dietary histidine to urocanate [35], a metabolite that is highly correlated with hepatic steatosis in mice [36]. Based on these findings, we tested whether histidine supplementation could rescue lipid accumulation in *Atg2*-*deficient Drosophila*. Dietary histidine administration (1–4 g/L) normalized lipid droplet size in *Atg2* RNAi midguts in a dose-dependent manner (Fig. S3A-B). Notably, 16S rRNA sequencing revealed that *Atg2* depletion induced marked microbial shifts, including reductions in *Actinobacteria*, *Cyanobacteria*, and *Muribaculaceae*, taxa previously implicated in histidine metabolism (Fig.S3C-D). Low-dose histidine supplementation (1 g/L) not only attenuated lipid accumulation but also extended the median lifespan in *Atg2*-deficient flies (Fig. S3E), suggesting that the therapeutic effects of histidine involve the modulation of microbiota-mediated metabolic pathways. These results establish that Atg2 regulates lipid homeostasis and longevity through histidine metabolism. To investigate the potential epigenetic role of histidine, we specifically quantified the expression of *HDAC3*, a key epigenetic regulator. Histidine supplementation restored *HDAC3* expression in the mutants (Fig. S3F), mechanistically linking the metabolic rescue by histidine to the reinstatement of this epigenetic regulator and strongly suggesting that its beneficial effect involves normalization of the epigenetic landscape.

### Atg2 governs microbiota‒epigenome crosstalk to orchestrate intestinal immune homeostasis

**Fig. 5:**
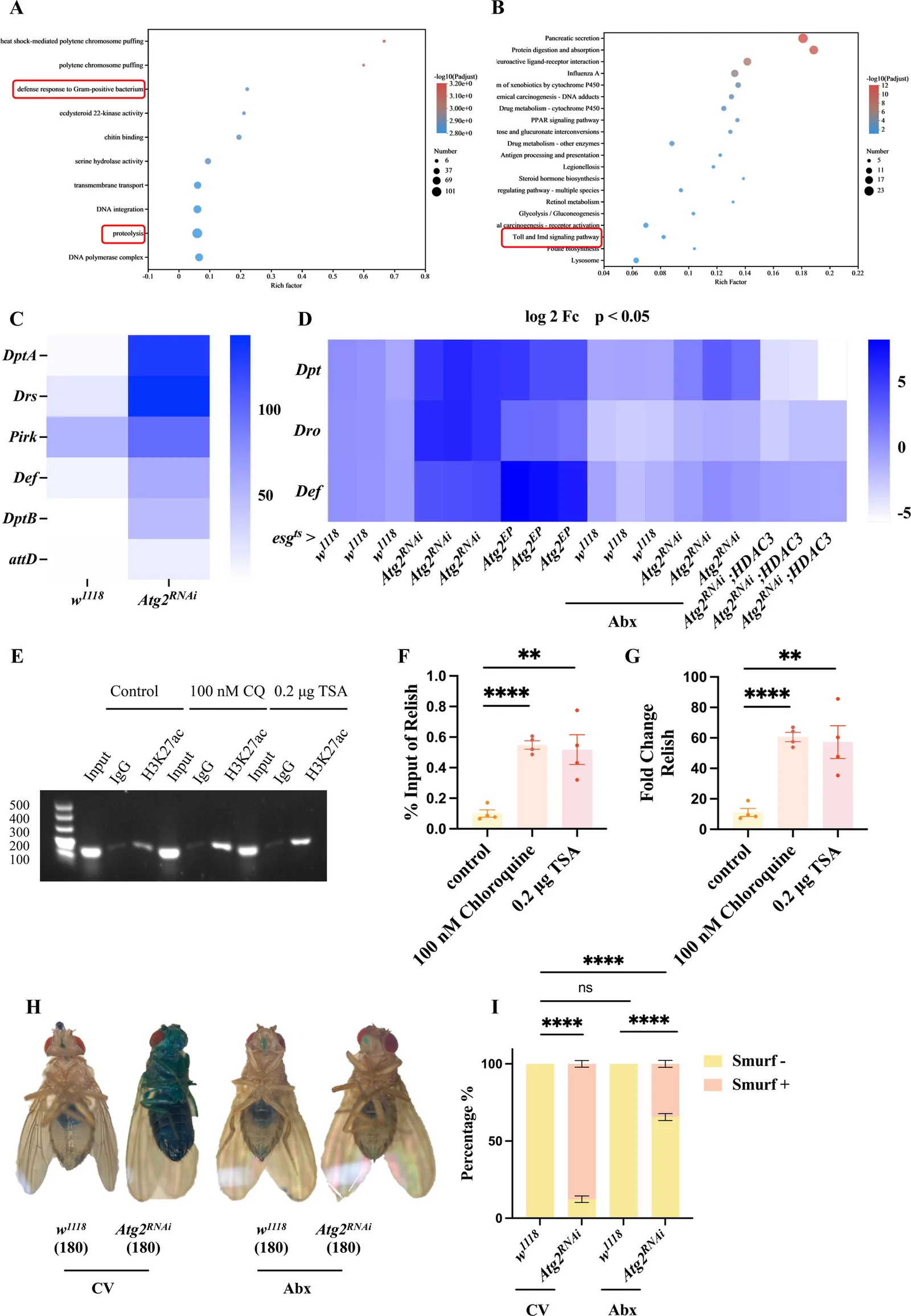
Atg2 triggers intestinal inflammation in a gut microbiota-dependent manner. **A-B** RNA-seq analysis of intestines from WT and *Atg2*-deficient flies from three independent experiments. **A** Gene Ontology biological process enrichment analysis of up- and downregulated genes. **B** KEGG pathway analysis highlighting significantly altered pathways. **C** Heatmap of immune signaling pathway‐associated genes. **D** RT‒qPCR analysis of the expression levels of AMP genes in the intestine from three independent experiments. **E–G** ChIP-PCR/qPCR analysis of H3K27ac enrichment at the *Relish* promoter locus from three independent experiments. **H-I** *Atg2* deficiency compromises intestinal barrier integrity in a microbiota-dependent manner. **H** Representative images of the Smurf assay from flies of the indicated genotypes under conventional or Abx-treated conditions. **I** Quantification of intestinal permeability. 30 flies per group from three independent replicates via chi-square tests with Bonferroni correction. The error bars represent the SEMs. Student’s t tests, *p < 0.05, **p < 0.01, ***p < 0.001, ****p < 0.0001, and NS (nonsignificant) represent *p* > 0.05

Emerging evidence has established gut dysbiosis as a potent activator of innate immune pathways, with recent studies demonstrating that microbiota-induced IMD/Toll signaling is involved in intestinal inflammation [37]. Quantitative microbiota profiling revealed a significant expansion of *Streptococcus* (Fig. S2A, S2C) in the gut microbiome, which was positively correlated with circulating biomarkers of systemic inflammation [38]. Our transcriptomic profiling of *Atg2*-deficient midguts revealed significant enrichment of IMD/Toll pathway components (Fig. 5A-B), accompanied by hyperactivation of Rel/NF-κB signaling (Fig. 5C).

Expression of the Rel/NF-κB pathway components *Diptericin* (*Dpt*), *Drosomycin* (*Dro*), and *Defensin* (*Def*) was markedly upregulated, as determined by RT–qPCR (Fig. 5D). To exclude potential off-target effects, we validated this immune activation in an independent *Atg2* mutant, which showed the same phenotype (Fig. 5D). This immune hyperactivation correlated with elevated antimicrobial peptide (AMP) expression, which was fully attenuated by Abx treatment (Fig. 5D).

By building upon established mechanisms whereby acetate modulates lipid metabolism through peptidoglycan receptor (PGRP)-LC-dependent activation of the IMD pathway [25, 40], we hypothesized that IMD/Toll pathway activation by acetate also depends on histone acetylation. We posited that microbial metabolite-driven immune activation requires chromatin accessibility mediated by histone acetylation. Notably, *HDAC3* overexpression, which rescues aberrant lipid metabolism caused by SCFAs dysregulation, concomitantly suppressed AMP gene transcription (Fig. 5D), demonstrating that microbiota-immune signaling is gated by epigenetic reprogramming. As a critical regulator of antibacterial defenses that directly controls AMP expression [39, 41], *Relish* represented a key target to assess for epigenetic reprogramming. Indeed, direct profiling of H3K27ac via ChIP-PCR/qPCR revealed robust enrichment at *Relish* promoters upon CQ and TSA treatment (Fig. 5E-G).

Consistent with IMD/Toll-mediated barrier dysfunction [39], *Atg2-*deficient flies presented severe intestinal permeability defects. Blue dye feeding assays revealed complete luminal retention in the control flies but systemic leakage in the *Atg2*-knockdown flies (Fig. 5H-I). Remarkably, Abx administration restored barrier integrity (Fig. 5H-I), confirming that microbiota-driven epithelial compromise occurred.

These findings delineate a hierarchical regulatory mechanism: Atg2 couples microbial SCFAs production to HDAC3-mediated chromatin compaction, thereby restraining the transcriptional activation of the IMD/Toll inflammatory pathways. This regulatory nexus positions Atg2 as an evolutionarily conserved integrator of microbial ecology and epigenetic control, dynamically balancing metabolic and immune homeostasis through histone modification landscapes.

### Atg2 regulates microbiota-dependent intestinal stem cell proliferation via epigenetic

Emerging evidence highlights the gut microbiota as a critical modulator of ISC dynamics [42]. Activation of the JNK, JAK/STAT, and EGFR pathways was observed following Atg2 suppression, which also enhanced ISC proliferation (Fig. S4).

**Fig. 6:**
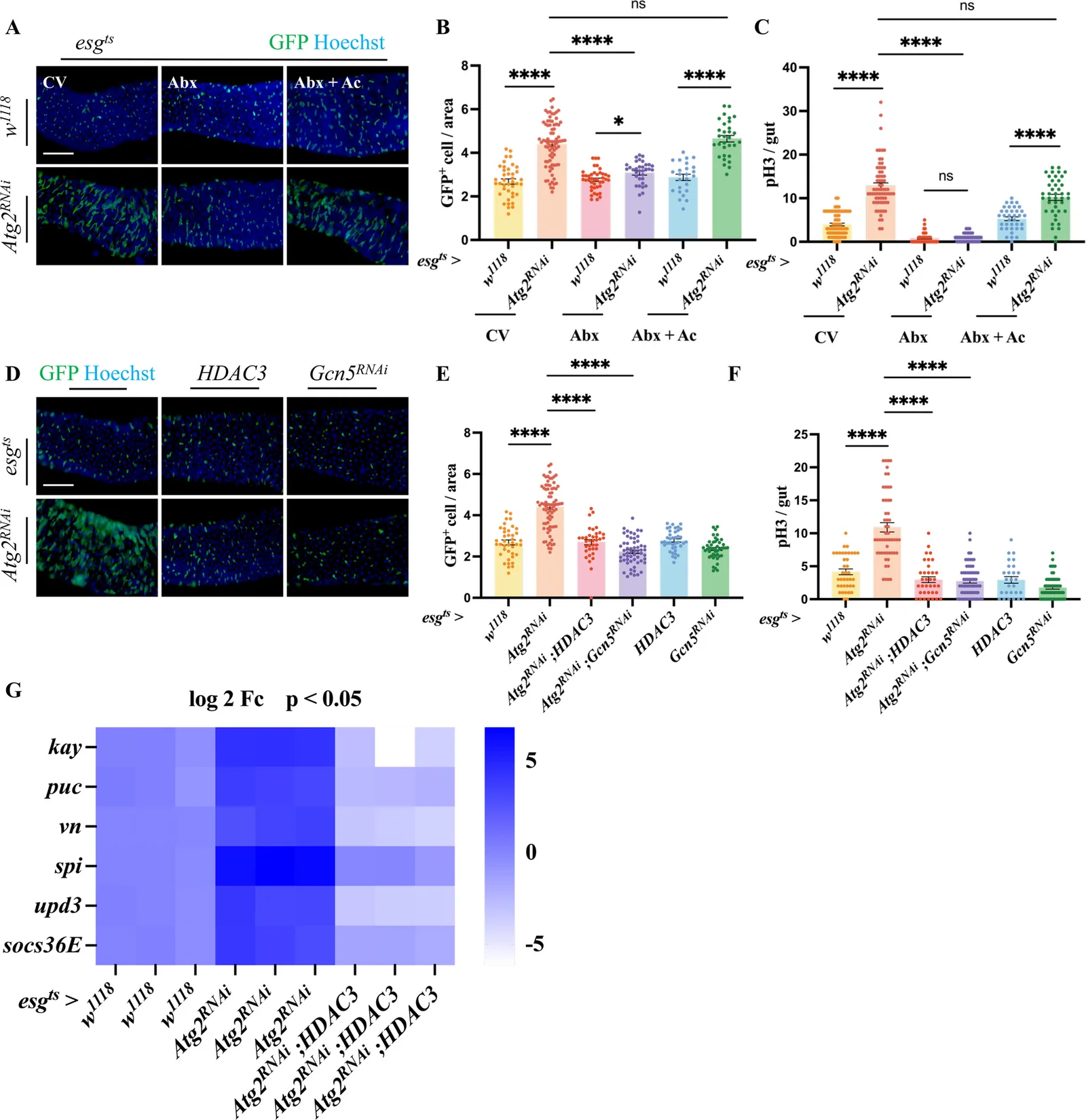
Atg2 regulates ISC proliferation in a gut microbiota-dependent manner. **A-B** Flies of the indicated genotypes were maintained under conventional (CV), antibiotic-treated (Abx), or acetate-supplemented (Abx + Ac) conditions for 7 days at 29°C. Midguts were immunostained for GFP and phospho-histone H3 (pH3), and counterstained with DAPI. ISC mitosis was quantified by pH3 + cell counting. **A** Representative image of the posterior midgut. **B-C** Quantification of GFP^+^ and pH3^+^ cells. Each point represents one fly from three biological replicates. **D-F** Progenitor-specific genetic manipulations driven by *esg*^*ts*^-*GAL4*. **D** Representative image of the posterior midgut. **E–F** Quantification of GFP^+^ and pH3^+^ cells. Each point represents one fly from three biological replicates. **G** RT‒qPCR analysis of EGFR, JNK, and JAK-STAT pathway gene expression in intestines following HDAC3 manipulation in progenitor cells from three biological replicates. Scale bars represent 50 μm. The error bars represent the SEMs. Student’s t tests, *p < 0.05, **p < 0.01, ***p < 0.001, ****p < 0.0001, and NS (nonsignificant) represent *p* > 0.05

This coordinated activation was confirmed by RT–qPCR, which showed upregulation of key components: *kay* and *puc* (JNK pathway), *upd3* and *Socs36E* (JAK/STAT pathway), and *Vein* and *Spitz* (EGFR pathway) (Fig. S4A). Genetic perturbation experiments confirmed the functional necessity of these pathways, as tissue-specific RNAi knockdown of EGFR, *JNK*, *or JAK/STAT* components reduced ISC hyperproliferation (Fig. S4B-C). Crucially, antibiotic treatment not only normalized ISC proliferation (Fig. 6A-C) but also attenuated pathway activation (Fig. S4A), demonstrating microbiota dependency.

Functional validation of microbial metabolites revealed that acetate supplementation (50 mM) restored ISC proliferation in Abx-treated *Atg2* RNAi midguts (Fig. 6A-C), mirroring the conserved SCFAs effects observed in murine intestinal organoids [43]. SCFAs function as potent epigenetic modulators across species, directly influencing chromatin states through histone acetyltransferase (HAT) activation and histone deacetylase (HDAC) inhibition in both *Drosophila* and murine models [10, 34]. Our mechanistic dissection revealed that SCFAs-driven hyperacetylation underlies pathological phenotypes in *Atg2*-deficient *Drosophila*. Knockdown of *Gcn5* completely rescued *Atg2* depletion-induced ISC hyperproliferation (Fig. 6D-F), demonstrating that aberrant HAT activity drives proliferative dysregulation. *HDAC3* overexpression normalized ISC proliferation rates (Fig. 6D-F) while suppressing *EGFR, JNK,* and *JAK/STAT* pathway expression (Fig. 6G). These findings establish a conserved regulatory axis in which microbial SCFAs modulate the HAT/HDAC balance to gatekeep proliferative signaling, with HDAC3 serving as a central rheostat for intestinal homeostasis.

## Discussion

Parallel findings in mammalian and *Drosophila* systems underscore the conservation of autophagy-mediated lipid regulation across species. Hepatic autophagy deficiency in mice drives excessive triglyceride and cholesterol accumulation [44], and our findings revealed that progenitor-specific *Atg2* depletion in *Drosophila* disrupts intestinal lipid homeostasis through a microbiota-SCFAs-epigenetic axis, mirroring the conserved mechanisms observed in mammalian systems.

Impaired autophagy contributes to the pathogenesis of both experimental models and human nonalcoholic steatohepatitis (NASH) [45]. Consistently, reduced autophagic flux has been observed in the livers of patients with nonalcoholic fatty liver disease (NAFLD) and NASH [46, 47]. Further supporting the role of autophagy in NAFLD progression, ATG2A expression is significantly altered in patient cohorts (GSE135251 [48]). Adipose tissue influences hepatic metabolism by releasing NEFAs and adipokines, which regulate lipid and glucose homeostasis [49]. In NAFLD, both elevated hepatic de novo lipogenesis (DNL) and increased NEFA flux from adipose tissue promote lipid accumulation in the liver [50]. In line with this, *Atg2* knockdown resulted in significant lipid deposition, as revealed by Nile Red staining (Fig. 1A-B, S1D-G) and elevated TG levels (Fig. 1C). Furthermore, NEFA levels were significantly elevated in *Atg2*-deficient flies (Fig. 1D). Strikingly, *Atg2* knockdown-induced lipid accumulation in *Drosophila* intestinal progenitors is strongly dependent on autophagy (Fig. 1A, S1J-M), in contrast with the findings of autophagy-independent lipid droplet regulation in transformed human cell lines [17]. This cell type-specific dichotomy likely arises from the unique metabolic demands of intestinal stem cells, which require robust autophagy for homeostasis [51], and the polarized lipid dynamics of epithelial tissues versus autonomous lipid handling in monolayer cultures. In support of the therapeutic potential of autophagy modulation, hepatic *Atg7* or *Atg14* overexpression reverses steatosis in obese mice [52, 53]. Hepatic *Atg12* deficiency drives NAFLD progression through impaired lipophagy [54, 55], mirroring our observation that *Atg1*/*8a*/*10*/*16* knockdown induces intestinal lipid deposition (Fig. S1L-M). Hepatic steatosis was also attenuated in this diet by the autophagy activator resveratrol [56]. These collective findings position autophagy enhancement as a viable strategy for treating lipid dysregulation disorders, bridging insights from invertebrate genetics to vertebrate pathophysiology.

Following rapamycin treatment in *Drosophila*, we observed a significant reduction in lipid droplet accumulation, as evidenced by restored Nile Red staining (Fig SH-I). Although rapamycin promotes early autophagy stages by inhibiting mTORC1, thereby activating the ULK1 complex and facilitating autophagosome nucleation and isolation membrane formation [57], this mechanism alone appears insufficient to explain the rescue observed in our model. Importantly, *Atg2* deficiency disrupts the core machinery required for autophagosome membrane expansion, preventing functional autophagosome completion even when autophagy initiation is enhanced by rapamycin. We therefore hypothesized that the observed lipid reduction may involve mechanisms independent of canonical autophagy. This hypothesis aligns with studies showing that rapamycin ameliorates obesity-related metabolic disorders in mammals via mTORC1 inhibition, acting through both autophagy-dependent and autophagy-independent pathways [46, 47, 58, 59]. The pleiotropic roles of mTORC1 in cellular metabolism, including regulation of growth, cytoskeletal remodeling, and immune responses [60], further support the potential contribution of non-autophagic mechanisms to lipid clearance.

Microbial SCFAs production may paradoxically fuel de novo lipogenesis in energy-overloaded states, contributing to excessive fat accumulation. Key lipogenic enzymes are upregulated in both *Atg2*-deficient flies (Fig. 1) and human hepatocellular carcinoma [61], with SREBP enhancing (Fig. 1J, S1A) their expression to promote lipid overproduction [62]. This pathological lipogenesis is further exacerbated by elevated Lsd-1 (Fig. 1G, S1A), a lipid droplet regulator implicated in hepatic steatosis [63–65].

The SCFAs-induced histone hyperacetylation observed in our model aligns mechanistically with that observed in mammalian systems. In mouse hepatocytes, acetate overproduction elevates FASN transcription via H3K9ac enrichment at its promoter [66]. Conversely, butyrate-mediated *HDAC3* suppression ameliorates obesity-associated pathologies, as demonstrated by reduced pancreatic inflammation and gut barrier dysfunction in high-fat diet-fed mice [67]. Paradoxically, while SCFAs administration promotes lipid accumulation under nutrient-replete conditions, *HDAC3* overexpression in our system normalizes FASN activation (Fig. 4B, D-G), mirroring hepatic steatosis phenotypes in *HDAC3* liver-specific knockout mice [68]. These findings position HDAC3 as a conserved rheostat balancing microbial metabolite signals and lipogenic gene expression.

*Drosophila melanogaster* serves as a powerful, genetically tractable model for intestinal inflammation research due to the evolutionary conservation of key intestinal features with mammals [69–71]. Importantly, the *Drosophila* gut epithelium, like its mammalian counterpart, relies on tight junctions and adherens junctions to maintain barrier integrity, functionally analogous to the mucus-barrier system [72]. Intestinal barrier permeability is a fundamental regulator of gut homeostasis, directly influencing the pathogenesis and progression of inflammatory bowel disease (IBD) [73]. Disruption of the epithelial barrier increases permeability [74], a hallmark phenotype observed in both IBD patients [74] and *Drosophila* IBD models [75]. Such barrier failure triggers intestinal inflammation and concomitant metabolic dysregulation [76]. Reinforcing the clinical relevance of barrier function, a genome-wide association study (GWAS) identified *ATG2* as a susceptibility locus for Crohn’s disease [77]. Consistently, we demonstrate that *Atg2* deficiency in *Drosophila* promotes immune dysregulation and exacerbates intestinal barrier dysfunction (Fig. 5), highlighting a conserved pathogenic mechanism. Emerging evidence highlights the conserved acetylation-dependent regulation of inflammatory pathways across species. In *Drosophila*, microbial-derived acetate activates the intestinal IMD pathway through protein acetylation [25]. Conversely, in mammals, HIPK2 suppresses NF-κB-driven inflammation by phosphorylating HDAC3, thereby preserving p65 acetylation status to dampen proinflammatory responses [78]. Our observation that *HDAC3* overexpression suppresses IMD/Toll signaling (Fig. 5D) aligns with human studies showing that suppressing HDACs blocks the activity of NF-κB, thereby inhibiting the downstream inflammatory signaling cascade [79]. The rescue of immune hyperactivation and lipid accumulation by antibiotics may represent a new layer of therapeutic mechanism for treating colitis-associated colorectal cancer and obesity.

Our study demonstrates that dietary histidine supplementation rescues *Atg2* deficiency-driven lipid accumulation in *Drosophila* through microbiota-dependent mechanisms. The dose-dependent reduction in lipid droplet size (Fig. S3A-B) is consistent with clinical evidence linking low circulating histidine levels to hepatic steatosis in both mammals and *Drosophila* [6]. Low-dose histidine extended lifespan in *Atg2*-deficient flies (Fig. S3E), an effect only partially attributable to lipid reduction. While histidine attenuated dysbiosis-associated lipogenesis, its interaction with the SCFAs-HDAC3 epigenetic hub remains to be fully elucidated. We propose that histidine may counteract SCFAs-driven hyperacetylation through two complementary mechanisms: direct competition for acetyl-CoA pools or enriching the microbiota that suppresses *Lactobacillus*-mediated SCFAs overproduction (Fig. 2D-E, S2D). Given its antioxidant and anti-inflammatory properties [80], histidine may mitigate immune hyperactivation (Fig. 5), potentially through microbiota-dependent suppression of IMD/NF-κB signaling. Together, these findings position histidine as a pleiotropic regulator of longevity under autophagy-impaired conditions. Moreover, the efficacy of nutritional-grade doses (1–4 g/L) mirrors human trials in which histidine improved NAFLD outcomes, highlighting its translational potential as a microbiota-targeted therapy for autophagy deficiency disorders. SCFAs promote ISC proliferation in mammalian systems, as demonstrated by enhanced regeneration in mice following butyrate administration [81], propionate-driven ISC expansion [82] and acetate-boosted IEC regeneration and differentiation [83]. Lactates produced by probiotics such as *Lactobacillus* increase the proliferation of Lgr5^+^ ISCs [84]. Consistent with these findings, we found that SCFAs supplementation led to ISC hyperproliferation in *Drosophila* (Fig. 6A-C). While SCFAs regulation of ISC dynamics has been established, microbiota-dependent epigenetic mechanisms remain poorly defined. Our study identified a conserved pathway in which microbial SCFAs induce acetylation of key signaling nodes—EGFR, JNK, and JAK/STAT—to activate their transcriptional programs (Fig. 6G, S4A). This acetylation-dependent epigenetic rewiring bridges microbial metabolite sensing with ISC proliferation.

While our *Drosophila* model elucidates fundamental mechanisms, key limitations must be addressed. Specifically, the absence of adaptive immunity in *Drosophila* precludes complete recapitulation of the intricate regulatory networks governing the human intestinal microenvironment. Furthermore, although our analysis focused on principal SCFAs (acetate, propionate, and butyrate), the gut microbiota produces a complex array of bioactive metabolites that may engage in synergistic or antagonistic crosstalk with SCFAs signaling. Finally, histidine acts primarily through microbial modulation or host signaling, which remains unclear, suggesting potential mechanisms warranting investigation in *Drosophila*.

## Conclusion

**Fig. 7:**
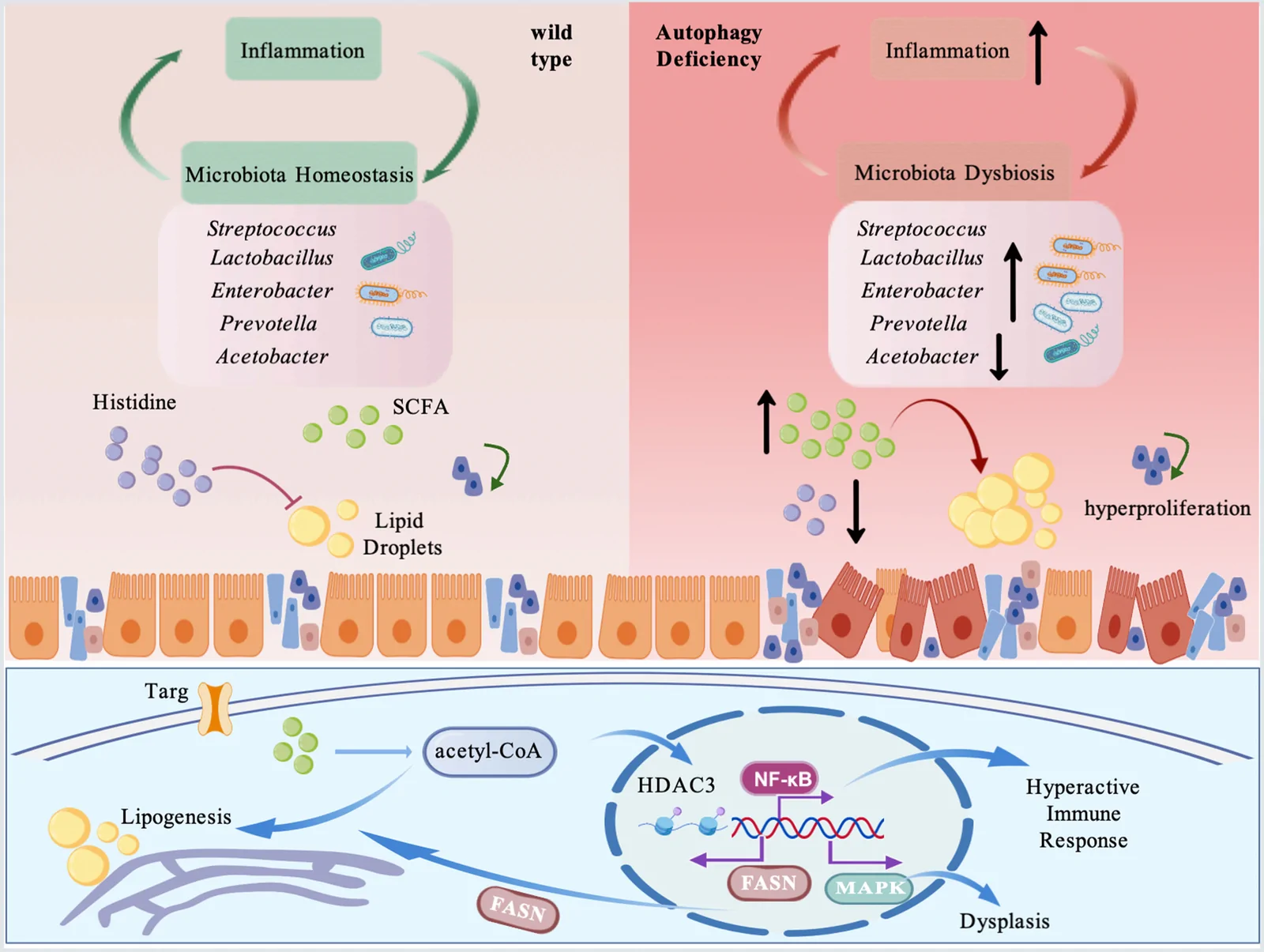
Model of Atg2 function in maintaining intestinal homeostasis. Atg2 loss in intestinal progenitors induces gut microbial dysbiosis. This dysbiosis increases SCFAs production while decreasing histidine levels. Excessive SCFAs disrupt acetyl-CoA metabolism, triggering widespread protein acetylation. This acetylation activates transcriptional programs that promote ectopic lipid deposition and immune activation. Concurrently, reduced histidine levels contribute to ectopic lipid deposition. Together, these alterations drive intestinal dysfunction and premature death

Our findings establish Atg2 as a central coordinator of microbial ecology, epigenetic reprogramming, and metabolic‒immune crosstalk—a paradigm increasingly relevant to human diseases (Fig. 7).

The striking overlap between *Drosophila* phenotypes and mammalian models of IBD/NAFLD underscores the translational value of invertebrate systems. By defining this autophagy‒microbiota‒metabolite regulatory axis, we propose a targeted therapeutic strategy: modulating microbial metabolites to correct autophagy-deficient metabolic pathologies while preserving the benefits of the commensal microbiota.

## Supplementary Information


Supplementary Material 1.

## Data Availability

The raw sequencing data generated in this study are publicly available in the Genome Sequence Archive (GSA) at the China National Center for Bioinformation (CNCB) under accession numbers: CRA026164 ( [https://ngdc.cncb.ac.cn/gsa/browse/CRA026164]) and CRA026189 ( [https://ngdc.cncb.ac.cn/gsa/browse/CRA026189]). All other data supporting the findings of this study are included in the published article and its Supplementary Information files. All other data supporting the findings of this study are included in the published article and its Supplementary Information files.
