# Correction: Atg2 coordinates microbial metabolite signaling and epigenetic remodeling to maintain intestinal lipid homeostasis in *Drosophila*

**DOI:** 10.1186/s40168-026-02512-8

**Published:** 2026-08-31

**Authors:** Ping Wang, Xinran Li, Jiangong Zhang, Jiewei Wang, Li Hua Jin

**Affiliations:** 1 College of Life Sciences, Northeast Forestry University, Harbin, China; 2 College of Food and Biological Engineering, Qiqihar University, Qiqihar, China

**Correction: Microbiome 14, 109 (2026)**

**https://doi.org/10.1186/s40168-026-02356-2**

Following publication of the original article [1], the author reported that there were assembly errors in the images of Figure 6D, Figure S1 and Figure S3, Specifically, Figure 6D: the same fluorescence image was inadvertently used for both the esgts; HDAC3 GFP (control) group and the esgts; tg2RNAi; Gcn5RNAi GFP (rescue) group. In Figure S1 and Figure S3: the same esgts; w1118 Nile Red control image was inadvertently used in both figures.

The incorrect Figure 6 is

**Figure:**
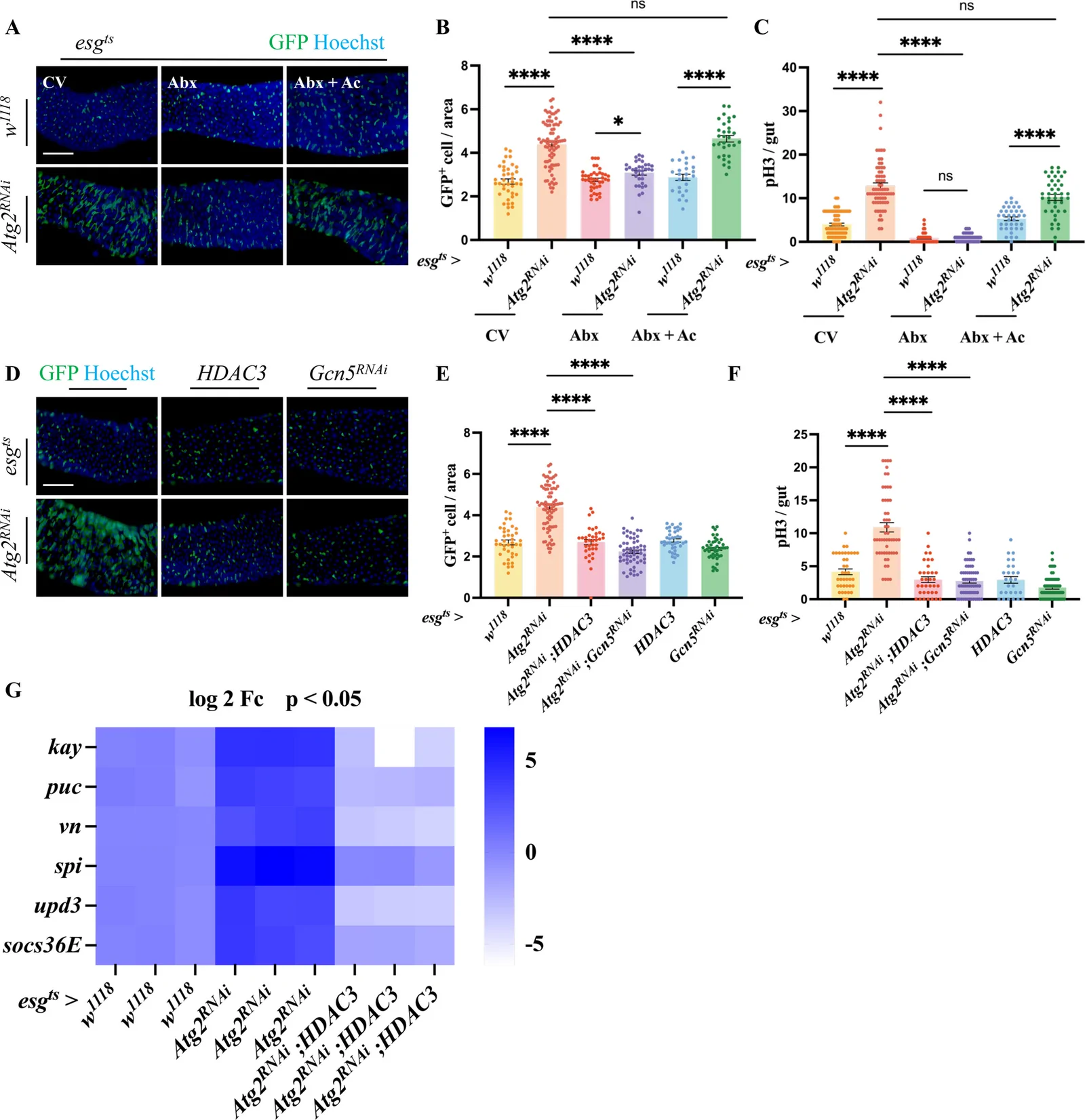

The correct Figure 6 is

**Figure:**
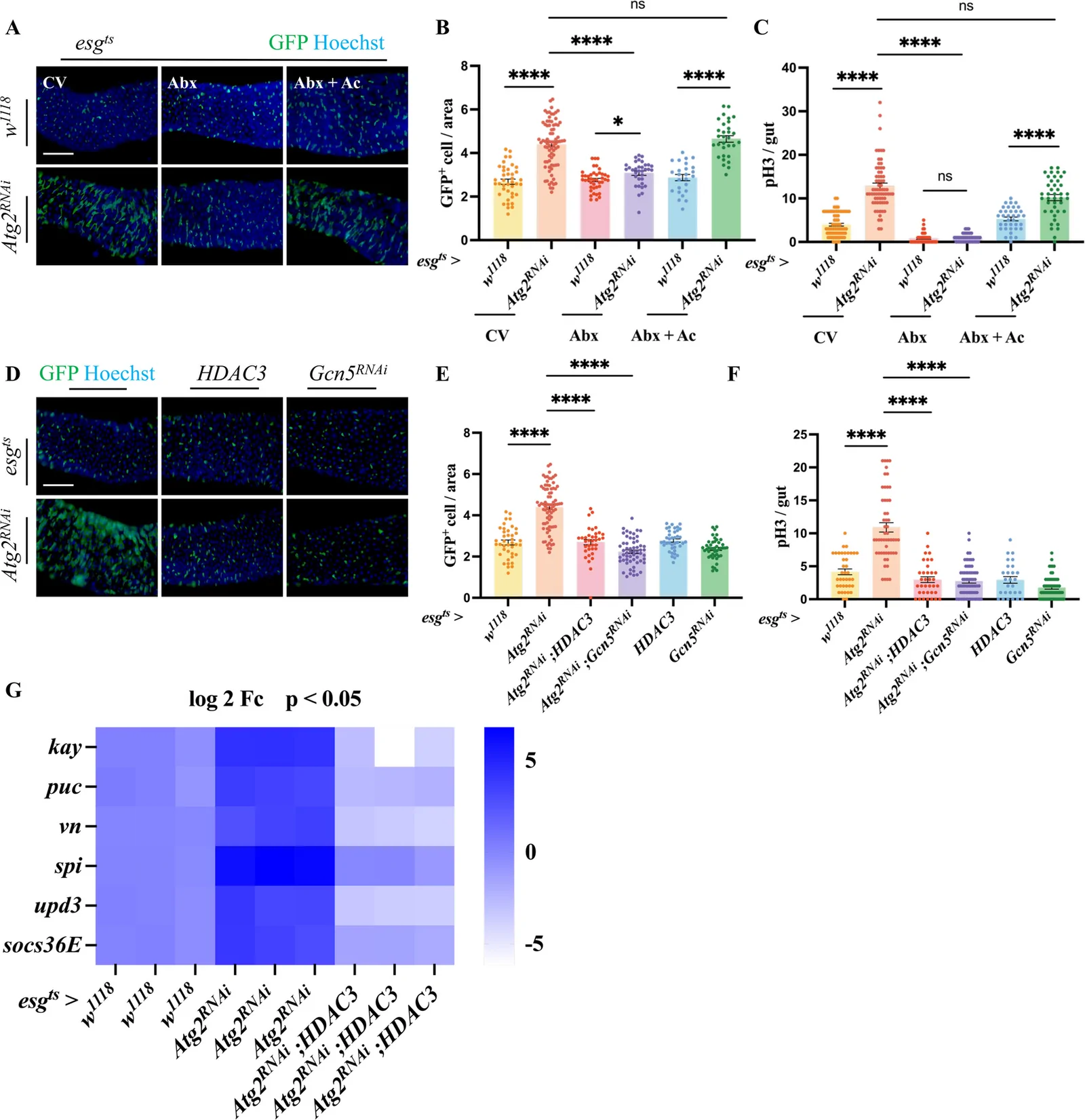

The incorrect Figure S3 is

**Figure:**
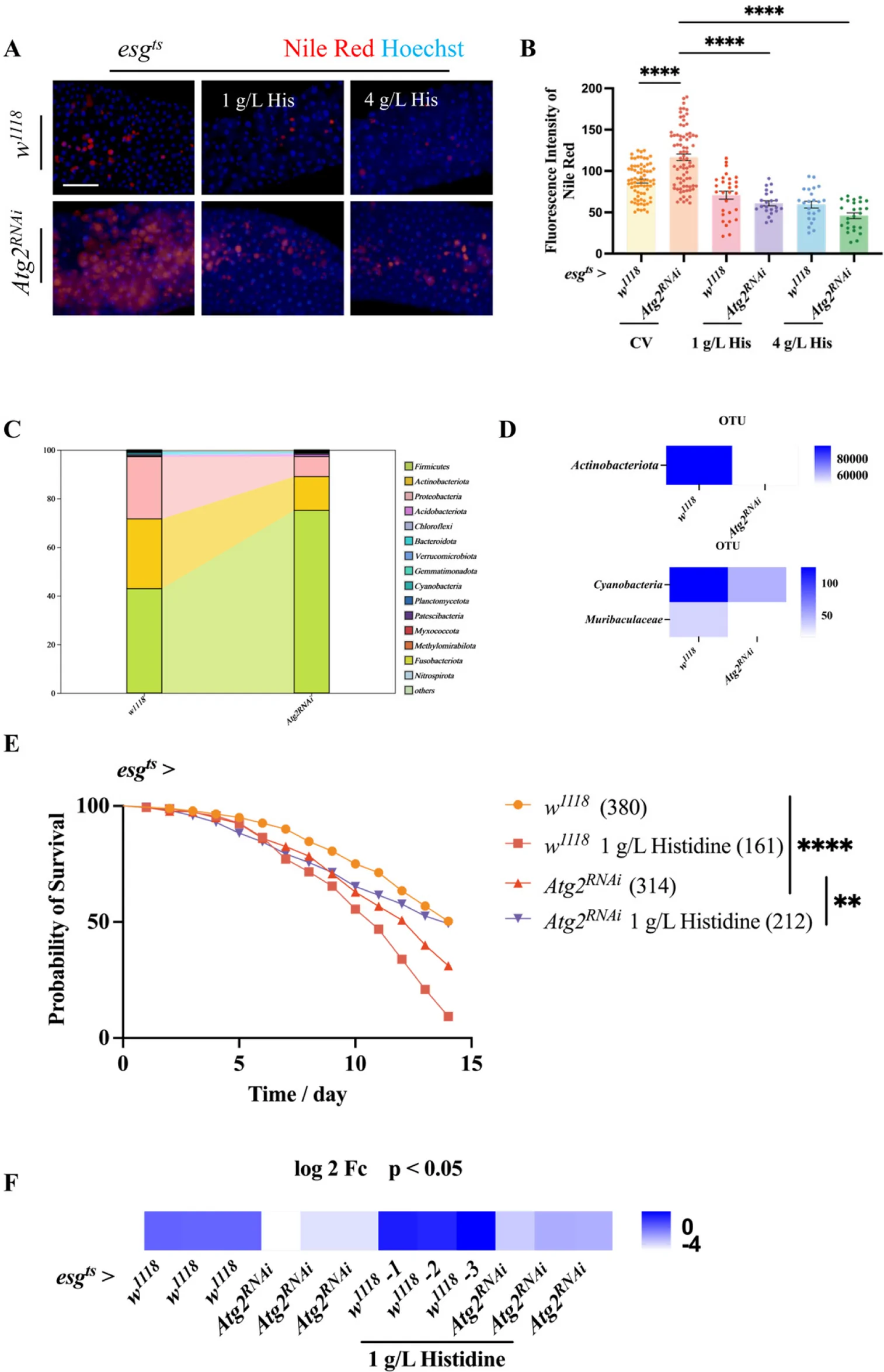

The correct Figure S3 is

**Figure:**
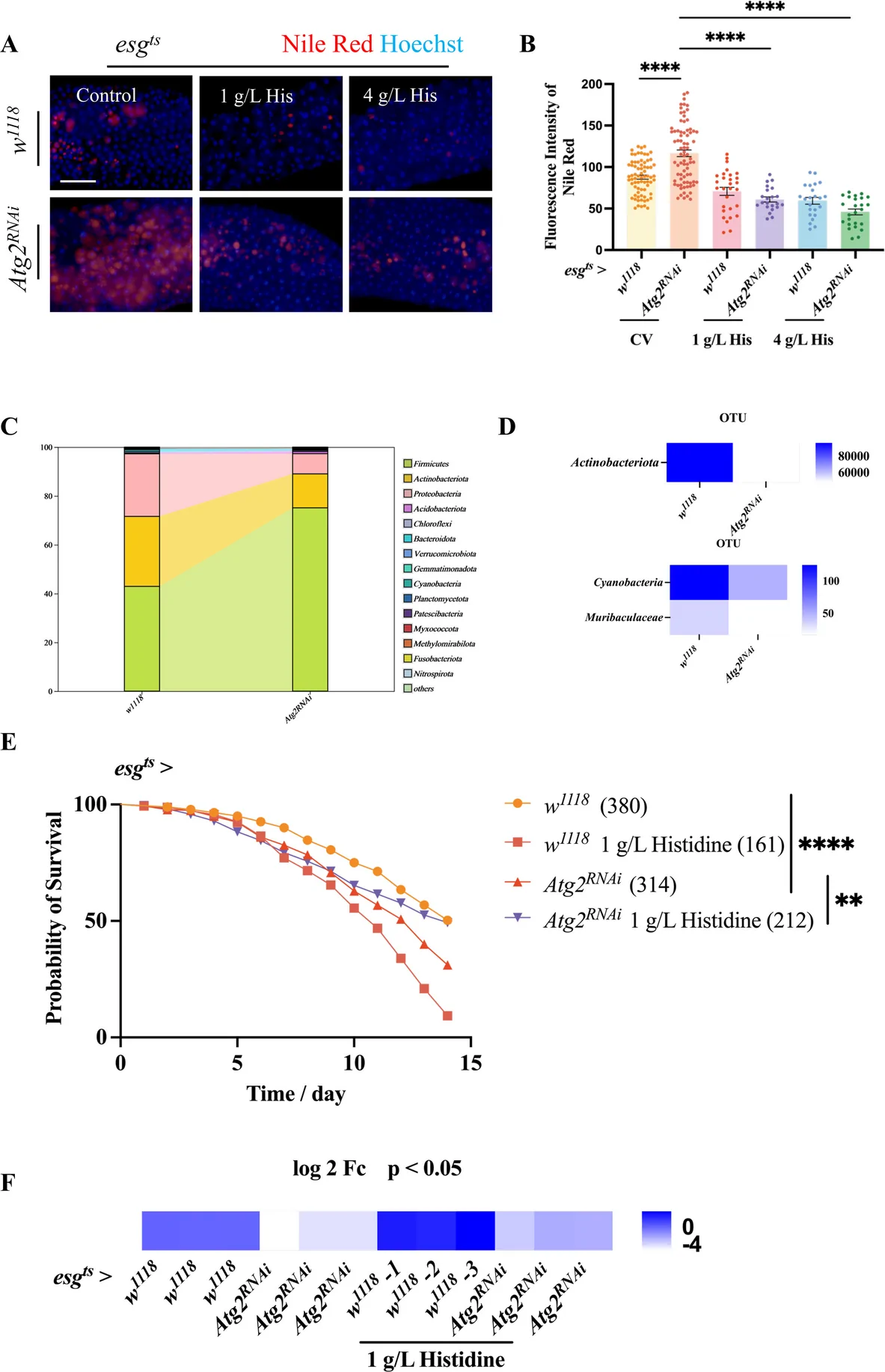

The original article has been updated.

## References

[1] Wang P, Li X, Zhang J, et al. Atg2 coordinates microbial metabolite signaling and epigenetic remodeling to maintain intestinal lipid homeostasis in *Drosophila*. Microbiome. 2026;14:109. 10.1186/s40168-026-02356-2. 41896985 PMC13063524

