# Supplementary material for: Atg2 coordinates microbial metabolite signaling and epigenetic remodeling to maintain intestinal lipid homeostasis in Drosophila
Source: Microbiome. 2026 Mar 27;14:109. doi: 10.1186/s40168-026-02356-2 (PMC13063524; doi:10.1186/s40168-026-02356-2)
Supplement: Supplementary file 2 — Supplementary Material 1. [file 40168_2026_2356_MOESM1_ESM.docx]

**Supplementary Information for**

**Atg2 coordinates microbial metabolite signaling and epigenetic remodeling to maintain intestinal lipid homeostasis in *Drosophila***

Ping Wang^1^, Xinran Li^1^, Jiangong Zhang^1^, Jiewei Wang^1, 2*^, Li Hua Jin^1*^

^*^ Corresponding author: Li Hua Jin and Jiewei Wang

**
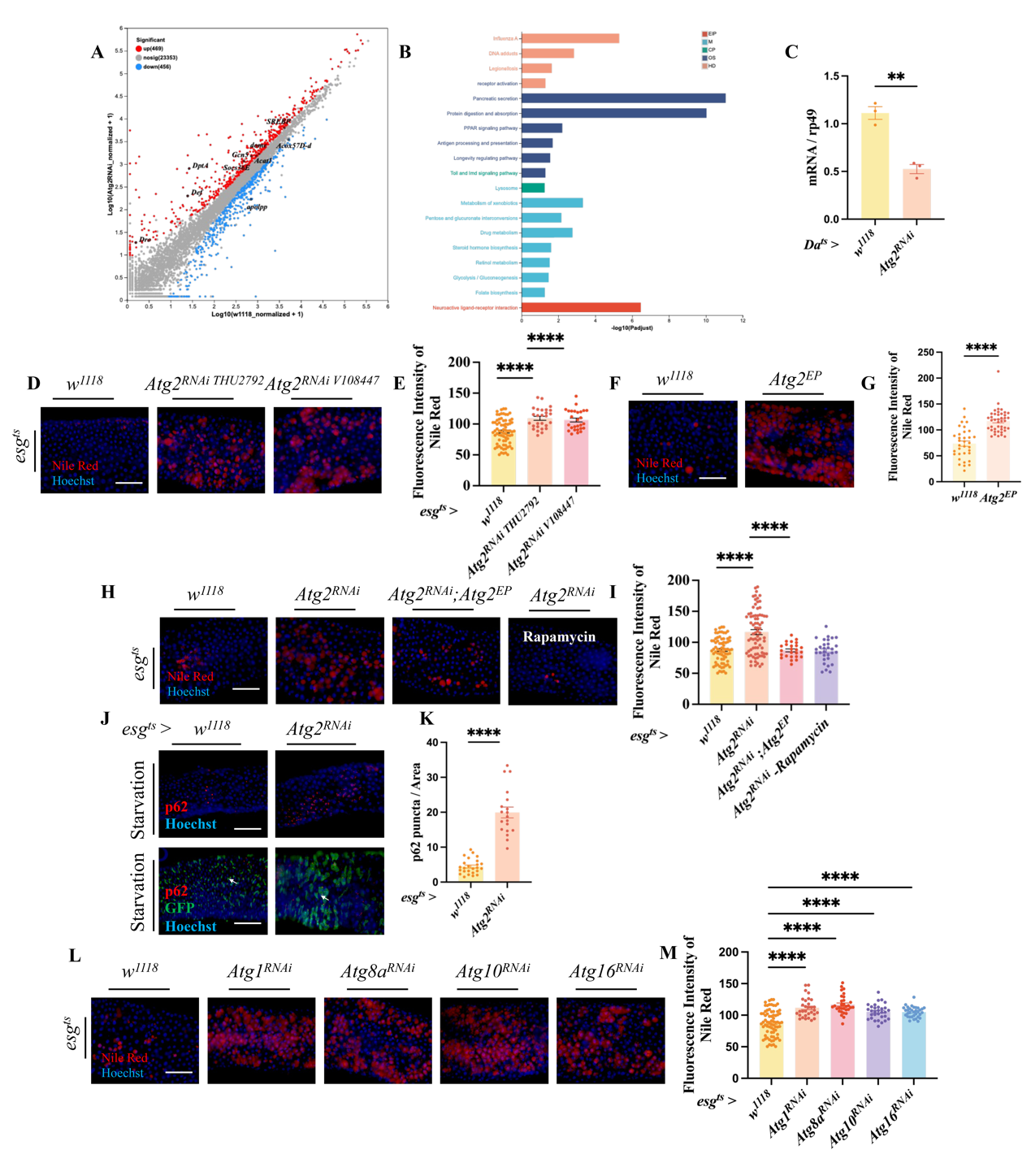
**

**Fig. S1** Autophagy regulates lipid homeostasis.

**A-B** RNA-seq analysis of intestines from WT and Atg2-deficient flies. Data are from three independent experiments. **A** Scatter plot depicting upregulated (red), downregulated (blue), and non-differentially expressed (gray) genes in Atg2-deficient flies compared to controls. **B** KEGG pathway analysis highlighting significantly altered pathways. **C** Validation of Atg2 knockdown via qPCR. Expression levels in Atg2 RNAi flies were normalized to the control. **D-E** Neutral lipid staining by Nile Red in control flies and those with Atg2 knockdown in intestinal stem cells/enteroblasts. **D** Representative view of the AMG. **E** Quantification of fluorescence intensity. **F-G** Nile Red staining of lipids in control and Atg2 mutant flies. **F** Representative view of the AMG. **G** Quantification of fluorescence intensity. **H-I** Overexpression of *Atg2* in progenitor cells driven by *esg^ts^-GAL4*. lipid content visualized by Nile Red. **H** Representative view of the AMG. **I** Quantification of fluorescence intensity. **J-K** Autophagy monitoring via p62 immunostaining. **J** Representative images showing p62 and GFP colocalization. **K** Quantification of p62 puncta. **L-M** Nile Red staining of neutral lipids in control and autophagy-deficient intestines. **L** Representative view of the AMG. **M** Quantification of fluorescence intensity Quantification of fluorescence intensity. Scale bars represent 50 μm. The error bars represent the SEMs. Student’s t tests, **p*< 0.05, ***p* < 0.01, ****p*< 0.001, *****p*< 0.0001, and NS (nonsignificant) represent *p*> 0.05. Each dot corresponds to one *Drosophila* from three biological replicates.

**
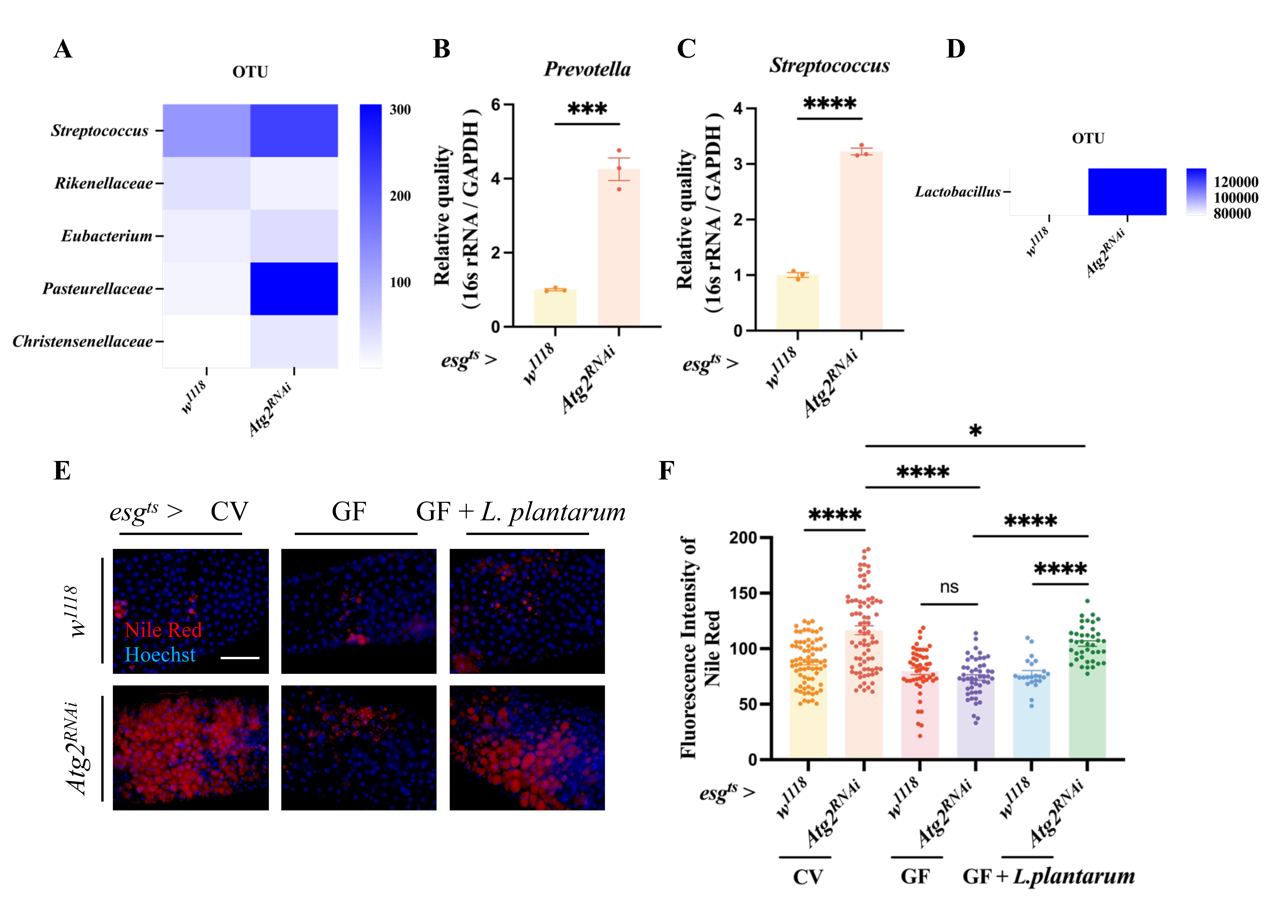
**

**Fig. S2** Atg2 deficiency alters the gut microbiota.

**A** Microbial composition (OTUs) in gut samples as determined by 16S rRNA sequencing. **B** Quantification of Prevotella abundance in the fly intestine by qPCR. Data are from three independent experiments. **C** Quantification of Streptococcus abundance in the fly intestine by qPCR. Data are from three independent experiments. **D** OTUs corresponding to *Lactobacillus* in gut samples analyzed via 16S rRNA sequencing. **E-F** Lipid staining with Nile Red in control and Atg2-deficient flies following bacterial monoassociation. **E** Representative view of the AMG. **F** Quantification of fluorescence intensity. Each dot corresponds to one *Drosophila* from three biological replicates. The error bars represent the SEMs. Student’s t tests, **p*< 0.05, ***p* < 0.01, ****p*< 0.001, *****p*< 0.0001, and NS (nonsignificant) represent *p*> 0.05.

**
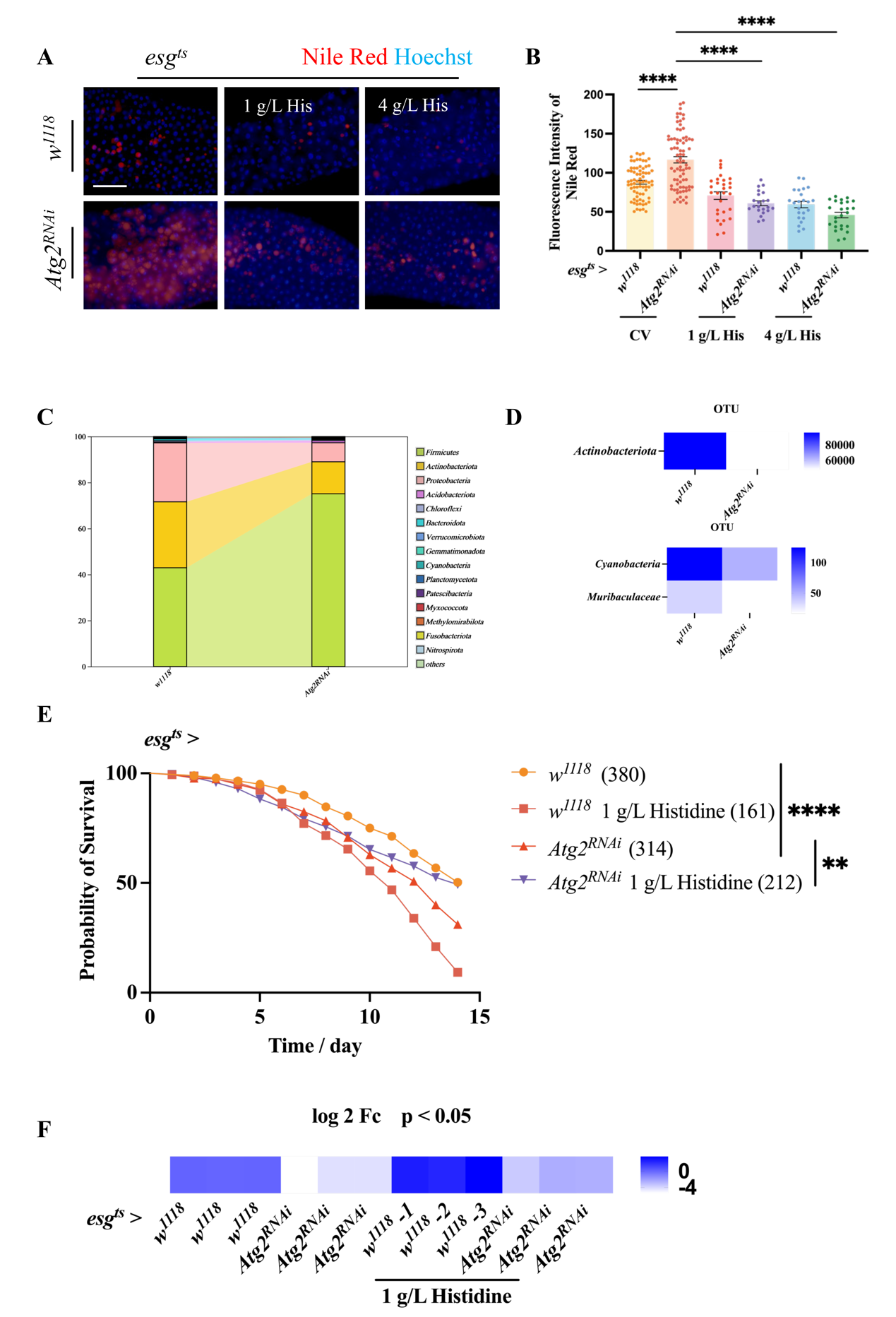
**

**Fig. S3** Histidine supplementation alleviates lipid accumulation in *Drosophila* models. **A-B** Effects of histidine supplementation on lipid droplets. Each dot corresponds to one *Drosophila* from three biological replicates. **A** Representative image of Nile Red staining in the AMG. **B** Quantification of fluorescence intensity. **C‒D** Atg2 deficiency alters the gut microbiota. **C** Relative abundance of the taxonomic classifications at the family level in intestinal samples. **D** OTUs in gut samples analyzed via 16S rRNA sequencing. **E** Lifespan comparison of control and Atg2-deficient flies under conventional and histidine-treated conditions (log-rank test). **F** RT‒qPCR analysis of the intestine revealed the expression levels of *HDAC3*. Data are from three independent experiments. Scale bars represent 50 μm. The error bars represent the SEMs. Student’s t tests, **p*< 0.05, ***p* < 0.01, ****p*< 0.001, *****p*< 0.0001, and NS (nonsignificant) represent *p*> 0.05.

**
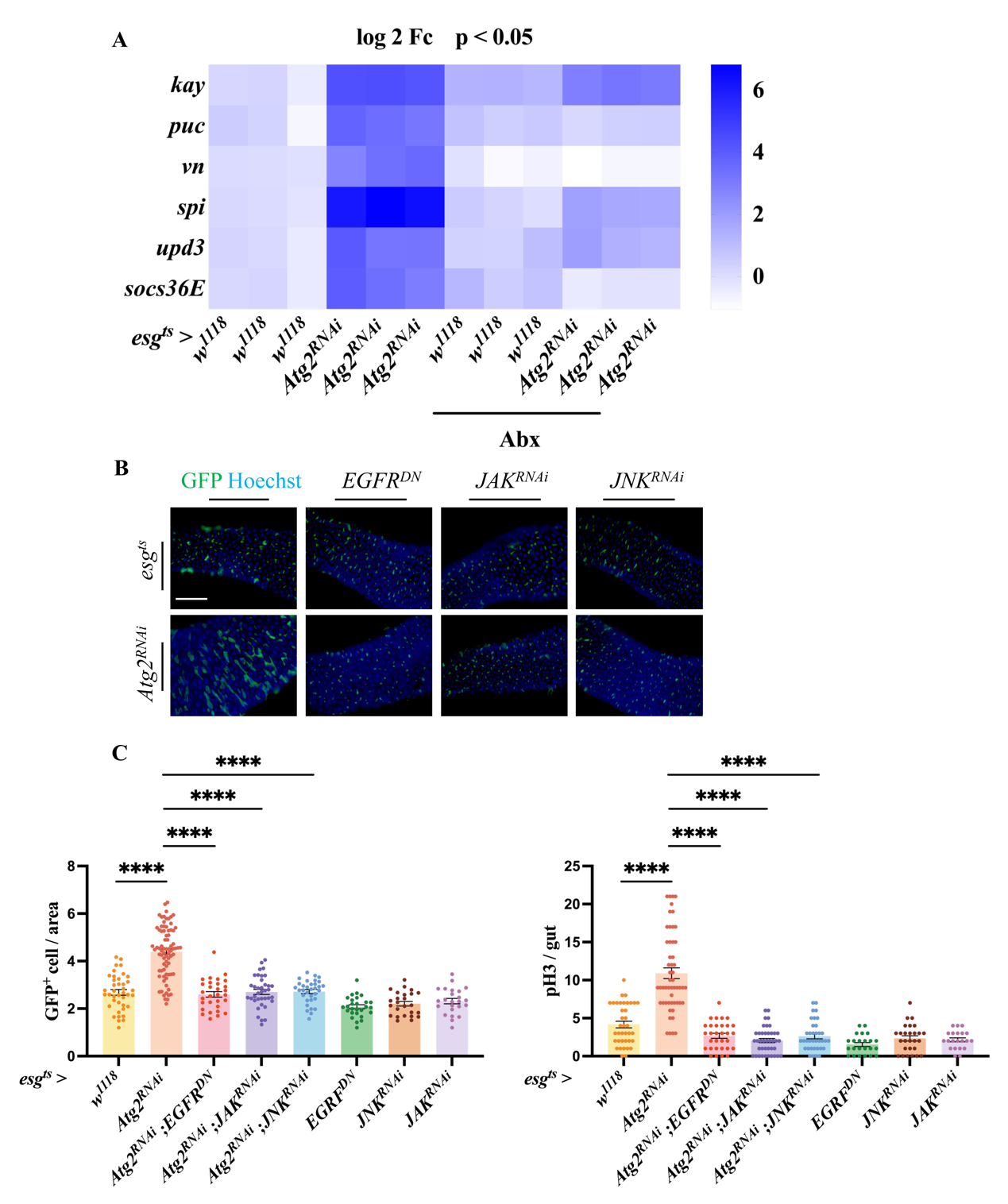
**

**Fig. S4** Atg2-mediated ISC mitoses by enhancing EGFR/JNK/JAK-STAT activity. **A** RT‒qPCR analysis of the intestine showing the expression levels of EGFR/JNK/JAK-STAT. Data are from three independent experiments. **B-C** Ectopic blockage of EGFR/JNK/JAK-STAT signaling via the expression of *EGFR^DN^*, *JNK^RNAi^*, and *JAK^RNAi^* in progenitors is sufficient to decrease the density of GFP+ and pH3^+^ cells, respectively. The midguts were stained with anti-GFP and anti-pH3 antibodies, as well as DAPI. ISC mitosis was quantified in pH3^+^ cells. Each dot corresponds to one *Drosophila* from three biological replicates. **B** Representative images of the posterior midgut. **C** Quantification of GFP^+^ and pH 3^+^ cells. Scale bars represent 50 μm. The error bars represent the SEMs. Student’s t tests, **p*< 0.05, ***p* < 0.01, ****p*< 0.001, *****p*< 0.0001, and NS (nonsignificant) represent *p*> 0.05.

Supplementary Table 1: *Drosophila* stock

| Name | origin |
| --- | --- |
| *w^1118^* | Tsinghua Fly Center |
| *esg^ts^GAL UAS GFP* | Tsinghua Fly Center |
| *Atg2*^RNAi^ THU3668 | Tsinghua Fly Center |
| *Atg2*^RNAi^ THU2792 | Vienne *Drosophila* RNAi Center |
| *Atg2*^RNAi^ V108447 | Vienne *Drosophila* RNAi Center |
| *Atg2^EP3697^* BDSC17156 | Bloomington Stock Center |
| *ACLY^RNAi^* BDSC65175 | Bloomington Stock Center |
| *ACSS^RNAi^* THU4288 | Tsinghua Fly Center |
| *Targ^RNAi^* BDSC57427 | Bloomington Stock Center |
| *HDAC1* BDSC32241 | Bloomington Stock Center |
| *HDAC3* BDSC55078 | Bloomington Stock Center |
| *HDAC6* BDSC5118*1* | Bloomington Stock Center |
| *Gcn5^RNAi^* THU1324 | Tsinghua Fly Center |
| *EGFR^DN^* | Tsinghua Fly Center |
| *JAK^RNAi^* | Tsinghua Fly Center |
| *JNK^RNAi^* | Tsinghua Fly Center |

Supplementary Table 2: Sequence information for RT-qPCR

| Name | Sequencing |
| --- | --- |
| *Rp49 F* | TACAGGCCCAAGATCGTGAAG |
| *Rp49 R* | GACGCACTCTGTTGTCGATACC |
| *Drs F* | CTTGTTCGCCCTCTTCGCTGTC |
| *Drs R* | AGCACTTCAGACTGGGGCTGCA |
| *Def F* | CGCTTTTGCTCTGCTTGCTTGC |
| *Def R* | TAGGTCGCATGTGGCTCGCTTC |
| *Dpt F* | ATGCAGTTCACCATTGCCGTC |
| *Dpt R* | TCCAGCTCGGTTCTGAGTTG |
| *Kay F* | CAGCATCAGCGACAGGATTA |
| *Kay R* | TCTGGCCGGTCTCAAAGTT |
| *Puc F* | CGTCATCATCAACGGCAAT |
| *Puc R* | AGGCGGGGTGTGTTTCTAT |
| *Vn F* | ACGCCAAGATCGACAAGGT |
| *Vn R* | TCCAGCATCGTAGTCGTCCA |
| *Spitz F* | GCGGGTGTTTTTGTTGTCAT |
| *Spitz R* | TTGGAATCGGGTTTCTCTACA |
| *Upd3 F* | CCCAGCCAACGATTTTTATG |
| *Upd3 F* | TGTTACCGCTCCGGCTAC |
| *Socs36E F* | AAAAAGCCAGCAAACCAAAA |
| *Socs36E R* | AGGTGATGACCCATTGGAAG |
| *HDAC3 F* | CTTCCACAGCGACGAGTACA |
| *HDAC3 R* | CTTCGTATAGGCCACGGAAT |

Supplementary Table 3: Sequence information for Bacterial qPCR

| Name | Sequencing |
| --- | --- |
| *GAPDH F* | TAAATTCGACTCGACTCACGGT |
| *GAPDH R* | CTCCACCACATACTCGGCTC |
| *Universal F* | CCTACGGGNGGCWGCAG |
| *Universal R* | GGACTACHVGGGTWTCTAAT |
| *Acetobacter F* | TAGTGGCGGACGGGTGAGTA |
| *Acetobacter R* | AATCAAACGCAGGCTCCTCC |
| *L. plantarum F* | TGATCCTGGCTCAGGACGAA |
| *L. plantarum R* | TGCAAGCACCAATCAATACCA |
| *Prevotella F* | CGATCAGTAGGGGTTCTGAG |
| *Prevotella R* | CTTCAGCGTCAGTTGTGCTC |
| *Streptococcus F* | GGTGGTGGTGCTCTTAACCA |
| *Streptococcus R* | ACGTGTTGTTGCAGCTTTTGT |

Supplementary Table 4: Sequence information for ChIP-qPCR/PCR

| Name | Sequencing |
| --- | --- |
| *FASN F* | AAGGAATCCGTCGAGTGCAG |
| *FASN R* | TGTCAGCTGGGCTAGTAGGT |
| *ACSS F* | AGCAGGATGACGAATGTGGG |
| *ACSS R* | CCTCCACCTCGGCATTCTTT |
| *Relish F* | AAGAGAGGAACGGCGAAGTG |
| *Relish R* | ACTCACGCAGAAACAGCTGA |
